# Neurogenesis is disrupted in human hippocampal progenitor cells upon exposure to serum samples from hospitalized COVID-19 patients with neurological symptoms

**DOI:** 10.1038/s41380-022-01741-1

**Published:** 2022-10-05

**Authors:** Alessandra Borsini, Blair Merrick, Jonathan Edgeworth, Gargi Mandal, Deepak P. Srivastava, Anthony C. Vernon, Gaia Nebbia, Sandrine Thuret, Carmine M. Pariante

**Affiliations:** 1 Stress, Psychiatry and Immunology Laboratory, Institute of Psychiatry, Psychology and Neuroscience, Department of Psychological Medicine, King’s College London, London, UK; 2 Centre for Clinical Infection and Diagnostics Research, Guy’s and St Thomas’ NHS Foundation Trust, London, UK; 3 School of Immunology and Microbial Sciences, Guy’s and St Thomas’ NHS Foundation Trust, London, UK; 4 Department of Basic and Clinical Neuroscience, Institute of Psychiatry, Psychology and Neuroscience, King’s College London, London, UK; 5 MRC Centre for Neurodevelopmental Disorders, King’s College London, London, UK

**Keywords:** Molecular biology, Cell biology, Stem cells

## Abstract

Coronavirus disease 2019 (COVID-19), represents an enormous new threat to our healthcare system and particularly to the health of older adults. Although the respiratory symptoms of COVID-19 are well recognized, the neurological manifestations, and their underlying cellular and molecular mechanisms, have not been extensively studied yet. Our study is the first one to test the direct effect of serum from hospitalised COVID-19 patients on human hippocampal neurogenesis using a unique in vitro experimental assay with human hippocampal progenitor cells (HPC0A07/03 C). We identify the different molecular pathways activated by serum from COVID-19 patients with and without neurological symptoms (i.e., delirium), and their effects on neuronal proliferation, neurogenesis, and apoptosis. We collected serum sample twice, at time of hospital admission and approximately 5 days after hospitalization. We found that treatment with serum samples from COVID-19 patients with delirium (*n* = 18) decreased cell proliferation and neurogenesis, and increases apoptosis, when compared with serum samples of sex- and age-matched COVID-19 patients without delirium (*n* = 18). This effect was due to a higher concentration of interleukin 6 (IL6) in serum samples of patients with delirium (mean ± SD: 229.9 ± 79.1 pg/ml, vs. 32.5 ± 9.5 pg/ml in patients without delirium). Indeed, treatment of cells with an antibody against IL6 prevented the decreased cell proliferation and neurogenesis and the increased apoptosis. Moreover, increased concentration of IL6 in serum samples from delirium patients stimulated the hippocampal cells to produce IL12 and IL13, and treatment with an antibody against IL12 or IL13 also prevented the decreased cell proliferation and neurogenesis, and the increased apoptosis. Interestingly, treatment with the compounds commonly administered to acute COVID-19 patients (the Janus kinase inhibitors, baricitinib, ruxolitinib and tofacitinib) were able to restore normal cell viability, proliferation and neurogenesis by targeting the effects of IL12 and IL13. Overall, our results show that serum from COVID-19 patients with delirium can negatively affect hippocampal-dependent neurogenic processes, and that this effect is mediated by IL6-induced production of the downstream inflammatory cytokines IL12 and IL13, which are ultimately responsible for the detrimental cellular outcomes.

## Introduction

Severe acute respiratory syndrome coronavirus 2 (SARS-CoV-2), the novel coronavirus that causes coronavirus disease 2019 (COVID-19), represents an enormous new threat to our healthcare system and particularly to the health of older adults [1, 2]. Although the respiratory symptoms of COVID-19 are well recognized [3], the neurological manifestations and their underlying mechanisms have not been extensively studied yet. So far, evidence has shown that COVID-19 patients can develop several neurological symptoms during the acute disease, including headaches, dizziness, loss of smell, cognitive problems, and in some cases even delirium symptoms [4, 5]. In older people, delirium, with the associated severe cognitive disturbances, including confusion and altered level of attention and consciousness, is associated with adverse outcomes, including prolonged hospitalization and death, even in the absence of COVID-19 infection [6].

So far, studies indicate that 20–30% of COVID-19 patients will present with or develop delirium or mental status changes during the course of the disease, with rates of 60–70% in cases of severe illness, at all ages [7–9]. While admission in intensive therapy units (ITU) is itself associated with delirium symptoms [10], a sub-group of COVID-19 patients start to show these symptoms early on before hospitalisation [7, 8], suggesting that the infection itself, rather than the hospital or ITU admission, plays a role in the development and/or exacerbation of an already compromised neurological scenario, especially in older patients. However, the exact mechanisms underlying the association between COVID-19 and delirium (or other neurological symptoms) are still not well understood.

It is likely that a major contributing mechanism to the development of neurological symptoms in COVID-19 patients is a hyper-activated immune system. Indeed, the virus infection is now well-known for its ability to induce an over-reacting immune response (defined as “cytokine storm” syndrome) characterized by the production of multiple inflammatory cytokines and chemokines measurable in the periphery, including interleukin 2 (IL2), IL6, IL8, IL12, IL13, interferon-gamma (IFN-γ) and tumour necrosis factor-alpha (TNF-α) [11–13]. Once produced, these peripheral inflammatory cytokines can then penetrate the blood brain barrier (BBB) [14–16] and directly affect brain mechanisms and induce neurological symptoms affecting cognition, memory, alertness and emotional state, and leading to delirium [17]. Among these brain mechanisms, hippocampal neurogenesis is regarded as one of the most important cellular processes involved in the regulation of cognition [18–20]. However, which of the cytokines that are elevated by COVID-infection are also directly relevant to the development of neurological symptoms is currently unknown.

Using an in vitro model of human hippocampal progenitor cells, we have already demonstrated that treatment with high concentrations of IL6, similar to those found in peripheral blood of COVID-19 patients (~50-500 pg/ml) [12, 21], can reduce neurogenesis and increase apoptosis [22, 23]. Other proinflammatory cytokines, like IL1β and interferon-alpha (IFN-α), have similar effects in this cellular model [24–27]. Moreover, we have shown that treatment of the same cellular model with serum samples from patients receiving IFN-α-treatment for Hepatitis C also increases apoptosis and reduces neurogenesis [28], and that such changes are predictive of the development of IFN-α-induced depression. Of note, similarly to COVID-19 patients, patients receiving IFN-α treatment also can present with cognitive impairments, inattention, memory loss and confusion [29]. This therefore suggests that reduction of hippocampal neurogenesis and/or increased apoptosis is one of the mechanisms underpinning the inflammation-induced neurological symptoms in COVID-19 infected patients, and that using serum samples from affected patients in this cellular model might be a mechanistically valid way to understand these effects.

Here we use this unique, well-established [22–25, 30–36], in vitro model of human hippocampal progenitor cells to assess the direct effect of treatment with serum samples from COVID-19 patients on hippocampal neurogenesis, and to investigate the molecular pathways activated by serum from patients with or without delirium. In particular, we tested the hypothesis that treatment of human hippocampal progenitor cells with serum from delirium patients would decrease cell proliferation and neuronal differentiation, and would increase apoptosis, when compared with serum from patients without delirium. We also identified the candidate inflammatory mechanisms that are involved in these effects of serum from patients.

## Methods and materials

### Patients’ cohort

The study comprises serum samples collected from a total of 36 patients who were admitted to Guy’s and St Thomas’ NHS Foundation Trust during the first wave of the COVID-19 pandemic in UK (March-June 2020). Samples used in this study were collected at time of hospital admission (*Time point 1*) and during admission (~5 days after hospitalization; *Time point 2*). All patients (with and without clinical diagnosis of delirium) started to present COVID-19 symptoms ~5 days before hospitalisation. *N* = 18 patients presenting with delirium symptoms at the time of hospital admission were age- and sex-matched with *N* = 18 patients who did not present any symptoms of delirium, both at time of hospitalization and across disease progression (see Table 1). None of these patients have been admitted to ITU prior or at either time of samples collection. Delirium symptoms were documented in clinical notes by the treating physician around the time of blood collection (during admission, Time point 1; and ~5 days (range: 2–7 days) after hospitalization, Time point 2). In both groups, the majority of patients presented with two or more medical comorbidities, with hypertension and type 2 diabetes being the most frequently observed; there were no differences in respiratory symptoms severity, and survival rate, between the two groups (see Table 1). Levels of C reactive protein (CRP) in serum also did not significantly differ between the two groups, at both time points (group effect: F = 1.3, p = 0.2; time effect: F = 0.4, p = 0.8, see Table 1). The study was approved by the King’s College Hospital Research Ethics Committee (Ref: 20/SC/0310).

**Table 1:** Characteristics of COVID-19 patients with and without delirium.

	Delirium	Non Delirium	
	(*n* = 18)	(*n* = 18)	*p* value
Age (years)			
Mean ± SD	76.2 ± 14.7	75.4 ± 14.2	*p* = 0.9^*^
Gender			
Male	12 (66.7%)	11 (61.1%)	*p* = 0.7^**^
Severity score^#^			
Mean ± SD	1.2 ± 1.2	1.3±1.2	*p* = 0.9^*^
Survival			
Yes	13 (72.2%)	16 (88.9%)	*p* = 0.2^**^
Two or more comorbidities^##^			
Baseline	15 (83.3%)	14 (77.8%)	*p* = 0.3^**^
Hypertension			
Baseline	13 (72.2%)	12 (66.7%)	*p* *=* 0.7^**^
Type 2 diabetes			
Baseline	5 (27.8%)	8 (44.4%)	*p* = 0.3^**^
CRP *Time point 1*			
Mean ± SD	72 ± 53.9 mg/L	115.5 ± 94.4 mg/L	*p* = 0.2^*^
CRP *Time point 2*			
Mean ± SD	83.7 ± 88.4 mg/L	97.5 ± 102.7 mg/L	*p* *=* 0.9^*^

Demographics of the COVID-19 patients’ cohort. Legend: ^*^Mann–Whitney U-test; ^**^Chi squared test; ^**#**^Severity Score: 0 = Asymptomatic or no requirement for supplemental oxygen, 1 = Requirement for supplemental oxygen (fraction of inspired oxygen (FiO2) < 0.4) for at least 12 h, 2 = Requirement for supplemental oxygen (FiO2 ≥ 0.4) for at least 12 h, 3 = Requirement for supplemental oxygen (FiO2 > 0.6) for at least 12 h, and not a candidate for escalation above level one (ward-based) care, 4 = Requirement for supplemental oxygen (FiO2 > 0.8) and peripheral oxygen saturations <90% (with no history of type 2 respiratory failure (T2RF)) or <85% (with known T2RF) for at least 12 h, 5 = Requirement for ECMO; ^**##**^Comorbidities: hypertension, type 2 diabetes mellitus^,^ (paroxysmal) atrial fibrillation aortic stenosis, benign prostatic hypertrophy/ hyperplasia, benign paroxysmal positional vertigo, cancer, congestive cardiac failure, chronic kidney disease, chronic obstructive pulmonary disease, gastroesophageal reflux disease, ischaemic heart disease, monoclonal gammopathy of undetermined significance, osteoarthritis, osteoporosis, peripheral vascular disease, rheumatoid arthritis, transient ischaemic attack, venous thromboembolism.

### Cell culture

Multipotent human hippocampal progenitor cell line HPC0A07/03 C (provided by ReNeuron, Surrey, UK) was used [22–25, 30–34, 36]. This model has been previously validated using a hippocampal newborn neuron specific marker, Prospero homeobox protein 1 (Prox1) [31]. Cells were left to proliferate in Dulbecco’s Modified Eagle Medium: Nutrient Mixture F-12 (DMEM/F-12) media to which we added the growth factors epidermal growth factor (EGF), basic fibroblast growth factor (bFGF) and 4-hydroxytamoxifen (4-OHT). Differentiation was initiated by removal of the growth factors and 4-OHT. Detailed information on this cell line can be found in our previous publication [31].

### In vitro treatment with serum samples from COVID-19 patients with and without delirium

**Fig. 1:**
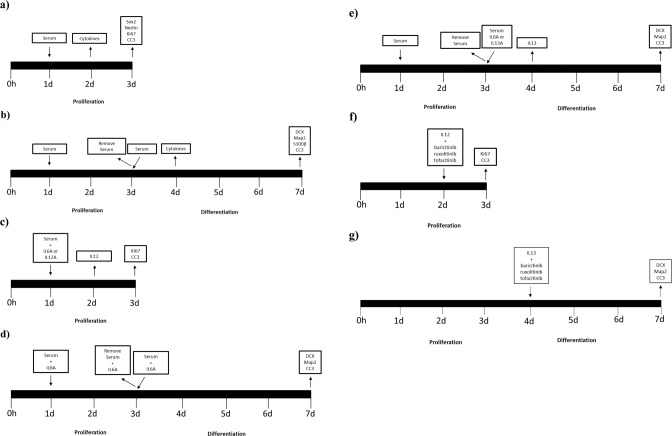
Timeline of in vitro experiments with the same serum samples. **a**–**e** Cells were treated with media containing 1% serum (from each COVID-19 patient, delirium and non-delirium) with or without IL6 Antibody (A) (0.1 μg/ml) or IL12A (0.3 μg/ml) for additional 2 days. At this stage cells were either fixed or left to differentiate for additional 4 days, again in presence of 1% serum (from the same COVID-19 patient previously used in the proliferation phase), with or without IL6A or IL13A (0.1 μg/ml). At the end of day 2 of proliferation and day 4 of differentiation, cell supernatant was collected for subsequent cytokines measurement. Legend: Sox2, sex determining region Y-box 2; DCX, doublecortin; Map2, microtubule-associated protein 2; S100β, S100 calcium-binding protein β; CC3, caspase 3; interleukin, IL.

Cells were plated on 96 well plates (Nunclon) at a density of 1.2 × 10^4^ cells per well. Following our established model [28], after 1 day of proliferation, cells were treated with media containing 1% serum (from each COVID-19 patient, delirium and non-delirium) and 0.5 mg/ml penicillin for 2 days. At this stage cells were either fixed with 4% PFA for 20 min at room temperature and immunostained, or left to differentiate for additional 4 days, again in presence of 1% serum (from the same COVID-19 patient previously used in the proliferation phase) and 0.5 mg/ml penicillin, and then again fixed and immunostained. In addition, at the end of 1 day of serum treatment during proliferation, and of 1 day of serum treatment during differentiation, cell supernatant was collected for subsequent cytokines measurement (Fig. 1a–d). For the experiment with the antibodies against cytokines, anti-IL6 antibody (0.1 μg/ml) was added during both proliferation and differentiation, anti-IL12 antibody (0.3ug/ml) during proliferation, and anti-IL13 antibody (0.1ug/ml) during differentiation (Fig. 1a–e), based on the results of supernatant cytokines measurement. Finally, for the experiments with the Janus kinase (JAK) inhibitors, baricitinib, ruxolitinib or tofacitinib (all 1 nM) were added during proliferation or differentiation together with recombinant IL12 or IL13 at concentrations previously found in supernatant of cells exposed to serum from patients with delirium (IL12: 20 pg/ml, IL13: 25 pg/ml) or without delirium (IL12: 3 pg/ml, IL13: 4 pg/ml) (Fig. 1f, g and Supplementary Fig. 5a–e).

### Immunocytochemistry and quantification of immunofluorescence

During the proliferation stage, fixed cells were stained for markers of stemness (sex determining region Y-box 2 (Sox2), Alexa 488 donkey anti-rabbit; 1:1000, Invitrogen; Nestin, Alexa 555 donkey anti-mouse, 1:1000, Invitrogen), proliferation (Ki67, Alexa 488 donkey anti-rabbit; 1:1000, Invitrogen) and apoptosis (CC3, Alexa 555 donkey anti-mouse, 1:1000, Invitrogen). During the differentiation stage, cells were stained for markers of neuroblasts and mature neurons (respectively, doublecortin (DCX), Alexa 555 donkey anti-rabbit; 1:1000, Invitrogen; microtubule-associated protein 2 (Map2), Alexa 488 donkey anti-mouse, 1:1000, Invitrogen), astrocytes (S100 calcium-binding protein β (S100β), Alexa 488 donkey anti-rabbit; 1:1000, Invitrogen), and again apoptosis (CC3; Alexa 555 donkey anti-rabbit, 1:1000, Invitrogen). All cells were labelled using DAPI dye, as in previous publications [22, 25, 28, 37–39]. The percentage of Sox2, Nestin, Ki67, DCX, Map2, S100β and CC3 positive cells over total DAPI positive cells was counted using an insight automated imaging platform (CellInsight) (Supplementary Fig. 1a–h for representative images). At least six independent experiments were conducted on independent biological cultures, and each sample was tested in quadruplicate.

### Multiplex cytokine measurement in cell supernatant and serum samples

Cell supernatants and serum samples from COVID-19 patients with and without delirium (collected both at *Time point 1* and *2*) were used for cytokines measurement (IL1β, IL2, IL4, IL6, IL8, IL10, IL12, IL13, TNF-α, IFN-γ), using the Human ProInflammatory Multiplex Very-Sensitive Kit from Meso Scale Discovery (MSD) (Gaithersburg, MD) and the SECTOR Imager MSD device, according to the manufacturers’ instructions. Detailed information on the cytokines analyses procedure can be found in our previous publication [22, 23, 40]. At least six independent experiments were conducted on independent biological cultures, and each sample was tested in duplicates.

### Statistical analysis

Statistical analyses were performed with IBM SPSS statistical software version 25, StataCorp STATA version 16 and GraphPad Prism version 8 and consisted of two-way mixed analysis of variance (ANOVA), Chi-square χ2 test, Mann–Whitney U-test, followed by Bonferroni’s post hoc analyses where appropriate. Variance was tested using the Shapiro–Wilk test. Data are presented as mean ± SD, and *p* values ≤ 0.05 were considered significant.

## Results

### Treatment with serum samples from COVID-19 patients with delirium decreased cell proliferation, neurogenesis, and increased apoptosis, when compared with serum from non-delirium patients

**Fig. 2:**
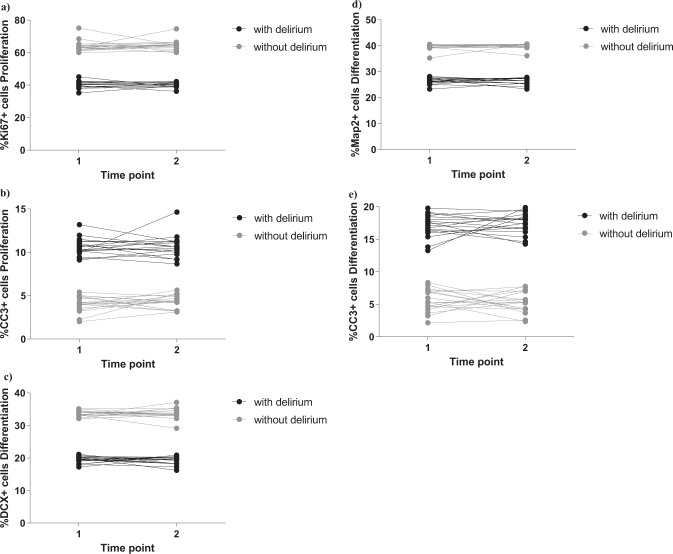
Cell treatment with serum samples from COVID-19 patients with delirium decreased cell proliferation, neurogenesis, and increased apoptosis, when compared with serum from non-delirium patients. **a**, **b** Cell treatment with serum samples from delirium patients decreased proliferation (KI67 + cells) and increased apoptosis (CC3 + cells), when compared with serum from non-delirium patients. There were no differences across Time point 1 and 2. **c**–**e** Treatment with serum samples from delirium patients decreased neurogenesis (DCX + and Map2+cells) and increased apoptosis (CC3 + cells), when compared with serum from non-delirium patients. Again, there were no differences across Time point 1 and 2. Two-way mixed ANOVA was performed.

As in our established protocol [28], we exposed cells to 2 days treatment during proliferation, with 1% serum sample from each COVID-19 patient with or without delirium, collected both at time of hospital admission (*Time point 1*) and during admission (*Time point 2*). We measured markers of cell proliferation (Ki67), apoptosis (CC3) and stemness (Sox2, Nestin) (Fig. 1a). Interestingly, we found that treatment of cells with serum samples from delirium patients decreased cell proliferation (group effect: *p* < 0.0001; time effect: *p* = 0.6, Fig. 2a and Supplementary Fig. 1i) and increased apoptosis (group effect: *p* < 0.0001; time effect: *p* = 0.5, Fig. 2b and Supplementary Fig. 1j), with no differences between cell treated with serum samples collected at *Time point 1* or *Time point 2*.

We did not observe any difference in the marker of stemness (Sox2 or Nestin), when comparing the two groups (delirium vs non-delirium) and the two time points (Supplementary Fig. 2a, b).

In the differentiation experiments, after the 2 days of treatment during proliferation, we left cells to differentiate for additional 4 days, again in presence of 1% serum. We measured markers of neurogenesis (DCX, Map2), astrogliogenesis (S100β) and apoptosis (CC3) (Fig. 1b). We found that treatment of cells with serum samples from delirium patients decreased neurogenesis (DCX: group effect: *p* < 0.0001; time effect: *p* = 0.9, Fig. 2c and Supplementary Fig. 1k; Map2: group effect: *p* < 0.0001; time effect: p = 0.9, Fig. 2d and Supplementary Fig. 1l) and increased apoptosis (group effect: *p* < 0.0001; time effect: *p* = 0.9, Fig. 2e and Supplementary Fig. 1m), again with no differences between the two time points.

Also, we did not observe any differences in the marker of astrogliogenesis (S100β), when comparing the two groups (delirium vs non-delirium) and the two time points (Supplementary Fig. 2c).

### Higher concentration of IL6 in serum samples from COVID-19 patients with delirium induces cells to produce IL12 and IL13, respectively during the proliferation and differentiation stage

In order to investigate the molecular mechanisms through which cell treatment with serum samples from delirium patients detrimentally affected cell proliferation, neurogenesis and apoptosis, we first measured levels of candidate cytokines (IL1β, IL2, IL4, IL6, IL8, IL10, IL12, IL13, TNF-α, IFN-γ) known to be modulated by the SARS-CoV-2 virus [11–13], in serum samples of both patients with delirium and without delirium.

**Fig. 3:**
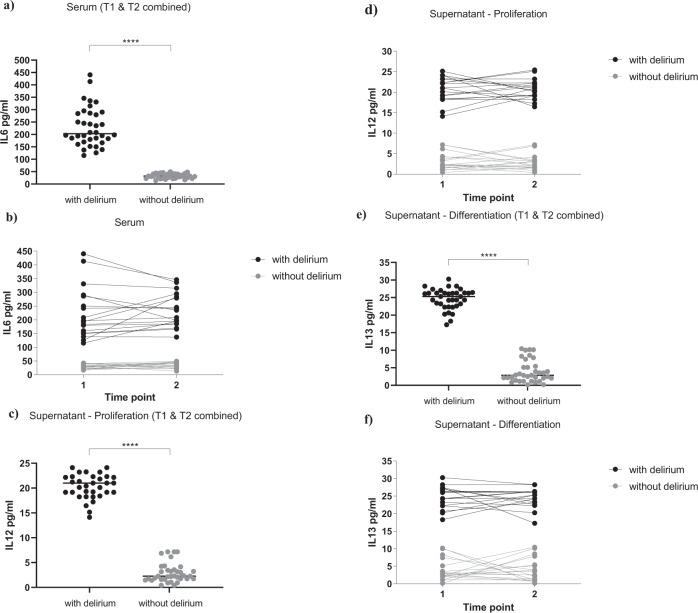
Higher concentration of IL6 in serum samples from COVID-19 patients with delirium induced cells to produce IL12 and IL13, respectively during the proliferation and differentiation stage. **a**, **b** Serum samples from delirium patients showed increased levels of IL6, when compared with non-delirium patients, both at Time point 1 and 2. **c**, **d** During proliferation, exposure of cells with serum samples from delirium patients (collected both at Time point 1 and 2) induced the production of IL12 in the supernatant, when compared with treatment with serum samples from non-delirium patients. **e**, **f** During differentiation, exposure of cells with serum samples from delirium patients (collected both at Time point 1 and 2) induced the production of IL13 in the supernatant, when compared with treatment with serum samples from non-delirium patients. Mann–Whitney U-test and two-way mixed ANOVA were performed. Data are shown as mean; ****p < 0.0001 comparisons as indicated.

Interestingly, we found a significantly higher concentration of IL6 in serum samples of patients with delirium (229.9 ± 79.1 pg/ml, Fig. 3a, b), when compared with serum samples from patients without delirium (32.5 ± 9.5 pg/ml, Fig. 3a, b), again with no differences between serum samples collected at *Time point 1* or *Time point 2* (group effect: *p* < 0.0001; time effect: *p* = 0.4, Fig. 3b). None of the other cytokines were differentially expressed between the two groups or the two time points (Supplementary Fig. 3a–i).

We then measured the same panel of cytokines in supernatant of cells exposed to treatment with serum samples from patients with or without delirium (collected both at *Time point 1* and *2*). In particular, cell supernatant was collected after 1 day of serum treatment during proliferation, and 1 day of serum treatment during differentiation (Fig. 1a, b).

Results showed significantly higher levels of IL12 during the proliferation, and IL13 during the differentiation, in the supernatant of cells treated with serum from delirium patients vs non-delirium patients (IL12: 20.6 ± 2.7 pg/ml vs 2.8 ± 2 pg/ml, Fig. 3c, d; IL13: 24.6 ± 3 pg/ml vs 3.9 ± 3.1 pg/ml, Fig. 3e, f), with no differences between supernatant of cells treated with serum samples collected at *Time point 1* or *Time point 2* (IL12: group effect: *p* < 0.0001; time effect: *p* = 0.8, Fig. 3d; IL13: group effect: *p* < 0.0001; time effect: *p* = 0.9, Fig. 3f).

Of note, concentrations of the same cytokines (IL12 and IL13) in the original serum samples were much lower, and did not differ between delirium and non-delirium patients (IL12: 2.1 ± 1 pg/ml vs 2.05 ± 1 pg/ml, Supplementary Fig. 3f; IL13: 2.7 ± 0.7 pg/ml vs 2.8 ± 1 pg/ml, Supplementary Fig. 3g), indicating that these differences in the supernatant levels are due to new production of these cytokines by the cells.

Finally, none of the other cytokines were differentially expressed in the supernatant of cells treated with serum samples from delirium or non-delirium patients, or from *Time point 1* or *Time point 2* (Supplementary Figure 3j-zz).

### During proliferation, treatment with an antibody against IL6 prevents the detrimental effect of serum from COVID-19 patients with delirium on both cell proliferation and apoptosis, and decreases IL12 production

In order to confirm that the detrimental effect of treatment with serum from delirium patients on cell proliferation and apoptosis was due to the higher levels of IL6 in the serum samples of the same patients, we exposed cells to treatment with serum from COVID-19 patients together with an antibody against IL6 (Fig. 1c).

**Fig. 4:**
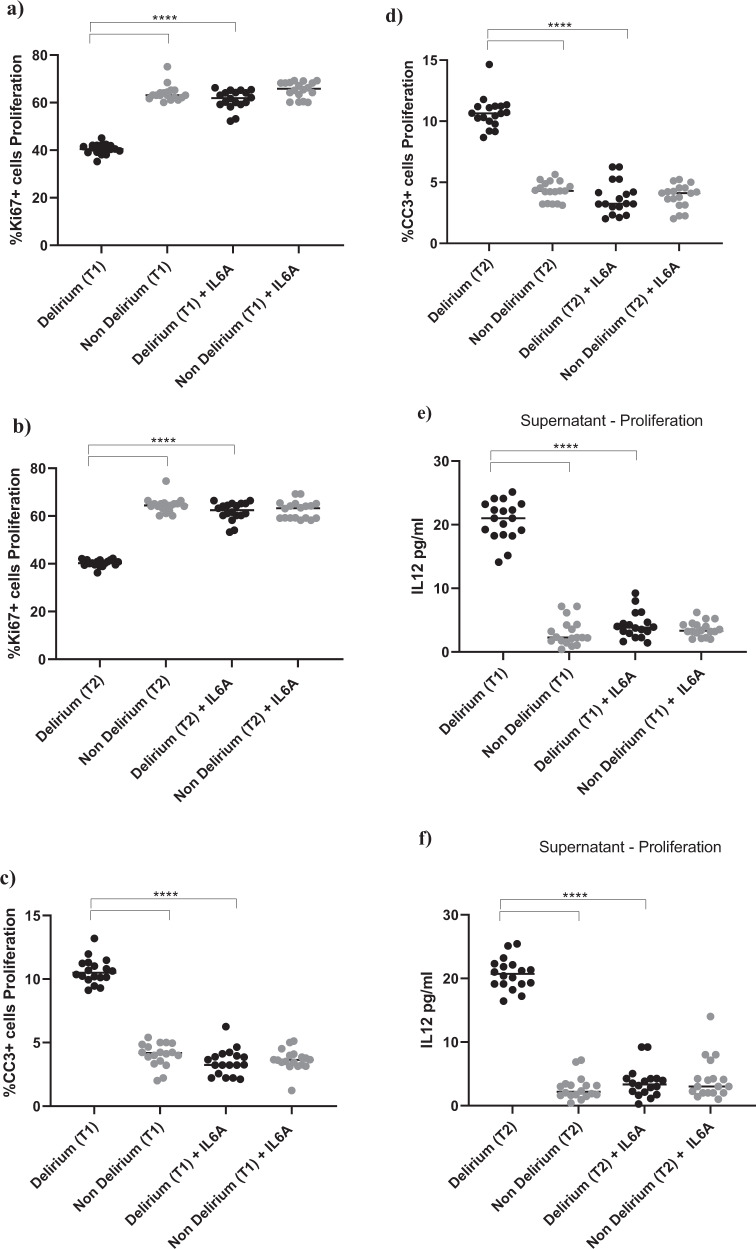

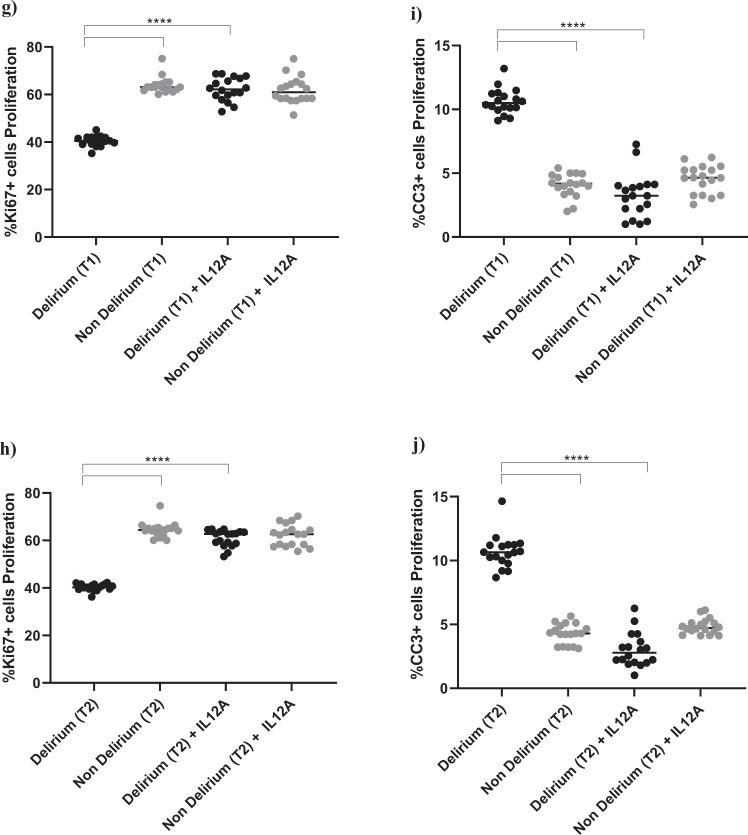
During proliferation, treatment with an antibody against IL6 prevented the detrimental effect of serum from COVID-19 patients with delirium on both cell proliferation and apoptosis and decreased IL12 production. **a**–**d** Cell treatment with IL6A (0.1 μg/ml) prevented the decrease in proliferation (Ki67+cells) and increase in apoptosis (CC3 + cells) previously observed upon treatment with serum samples from patients with delirium. This is confirmed across treatment with serum samples collected at Time point 1 and 2. **e**, **f** Treatment with IL6A also prevented production of IL12 in cell supernatant. **g**–**j** Cell treatment with IL12A (0.3 μg/ml) prevented the decrease in proliferation (Ki67+cells) and increase in apoptosis (CC3 + cells) previously observed upon treatment with serum samples from patients with delirium. Again, this is confirmed across treatment with serum samples collected at Time point 1 and 2. Two-way mixed ANOVA with Bonferroni’s post hoc test was performed. Data are shown as mean; ****p < 0.0001 comparisons as indicated.

Interestingly, treatment with IL6 antibody prevented the decrease in proliferation (Ki67) and increase in apoptosis (CC3) caused by treatment with serum from delirium patients, when compared with treatment with serum from non-delirium patients (*Time point 1*, delirium vs delirium + IL6 antibody; for Ki67, 40.2% vs 61.3%, *p* < 0.0001; for CC3, 10.2% vs. 3.4%, *p* < 0.0001), with no differences between cells treated with serum samples collected at *Time point 2* (Fig. 4a–d).

Moreover, treatment of cells with serum samples from delirium patients and IL6 antibody prevented the production of IL12 in cell supernatant (*Time point 1*, delirium vs delirium + IL6 antibody; IL12: 20.6 ± 2.7 pg/ml vs 4.1 ± 2.1 pg/ml, *p* < 0.0001,), again with no differences between *Time point 1* and *Time point 2* (Figs. 1c, 4e, f).

This finding seems to suggest that, during proliferation, the detrimental effect on cell proliferation and apoptosis caused by IL6, which is produced in higher concentrations in serum from delirium patients, may be mediated by the production of IL12 in the brain environment. Indeed, treatment of cells with an antibody against IL12 prevented the decrease in cell proliferation (Ki67) and increase in apoptosis (CC3) caused by treatment with serum from delirium patients (*Time point 1*, delirium vs delirium + IL12 antibody; for Ki67, 40.2% vs 62.2%, *p* < 0.0001; for CC3, 10.2% vs 3.2%, *p* < 0.0001). Again, there were no differences between cells treated with serum samples collected at *Time point 1* or *Time point 2* (Figs. 1c, 4g–j).

### During differentiation, treatment with an antibody against IL6 prevents the detrimental effect of serum from COVID-19 patients with delirium on both neurogenesis and apoptosis, and decreases IL13 production

In order to investigate whether the detrimental effect of treatment with serum from delirium patients on neurogenesis and apoptosis during *differentiation* was also mediated by a higher concentration of IL6 in the serum samples of the same patients, we exposed cells to treatment with serum and an antibody against IL6, first during proliferation, then during differentiation, and finally during both proliferation and differentiation (Fig. 1d, e).

**Fig. 5:**
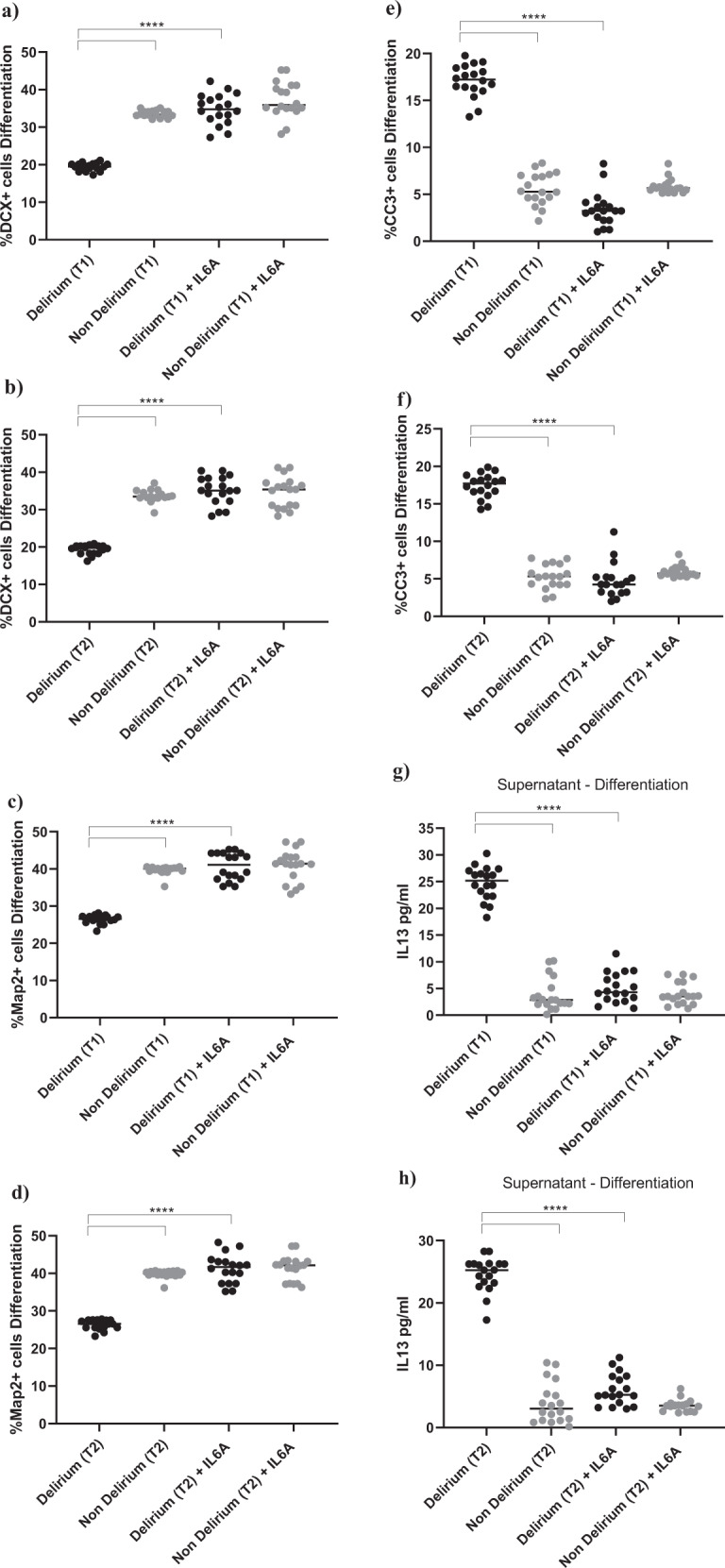

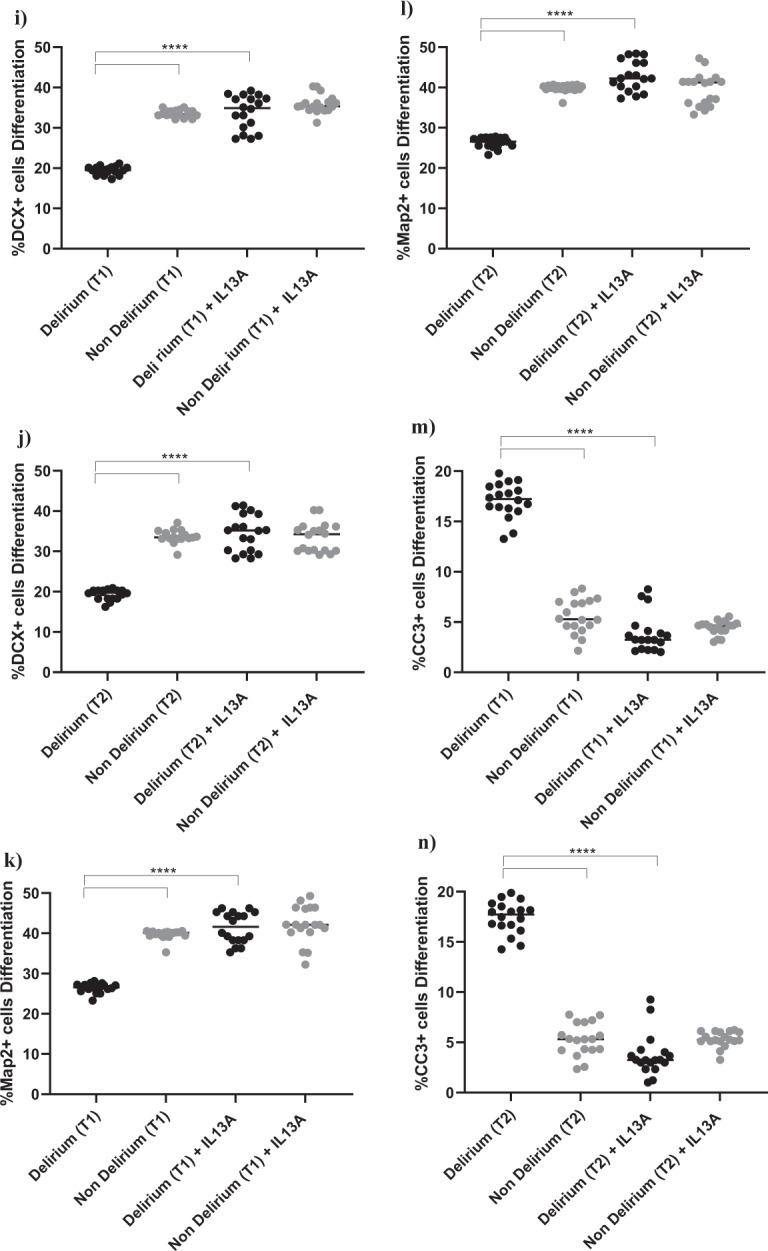
During differentiation, treatment with an antibody against IL6 prevented the detrimental effect of serum from COVID-19 patients with delirium on both neurogenesis and apoptosis and decreased IL13 production. **a**–**f** Cell treatment with IL6A (0.1 μg/ml) prevented the decrease in neurogenesis (DCX + and Map2+cells) and increase in apoptosis (CC3 + cells) previously observed upon treatment with serum samples from patients with delirium. This is confirmed across treatment with serum samples collected at Time point 1 and 2. **g**, **h** Treatment with IL6A also prevented production of IL13 in cell supernatant. **i**–**n** Cell treatment with IL13A (0.1ug/ml) prevented the decrease in neurogenesis (DCX + and Map2+cells) and increase in apoptosis (CC3 + cells) previously observed upon treatment with serum samples from patients with delirium. Again, this is confirmed across treatment with serum samples collected at Time point 1 and 2. Two-way mixed ANOVA with Bonferroni’s post hoc test was performed. Data are shown as mean; ****p < 0.0001 comparisons as indicated.

Exposing cells to IL6 antibody during the proliferation stage did not prevent the effect of serum on the aforementioned cellular outcomes (Supplementary Fig. 4a–f). However, treatment of cells with IL6 antibody during the differentiation stage prevented the reduction in neurogenesis (DCX, Map2) and increase in apoptosis (CC3) previously observed upon treatment with serum from delirium patients, when compared with serum from non-delirium patients (*Time point 1*, delirium vs delirium + IL6 antibody; for DCX, 19.1% vs 34.8%, *p* < 0.0001; for Map2, 25.1% vs 40.7%, *p* < 0.0001; for CC3, 17.3% vs 3.4%, *p* < 0.0001). Again, there were no differences between cells *Time point 1* and *Time point 2* (Fig. 5a–f). Results were confirmed when exposing cells to IL6 antibody during both the proliferation and differentiation stage (Supplementary Fig. 4g–l).

Moreover, similar to the effects of IL6 antibody during the proliferation phase on the production of IL12, treatment of cells with serum samples from delirium patients and IL6 antibody during the differentiation stage prevented the production of IL13 in cell supernatant, (*Time point 1*, delirium vs delirium + IL6 antibody; IL13: 24.6 ± 3 pg/ml vs 5.1 ± 2.7 pg/ml, *p* < 0.0001,), again with no differences between *Time point 1* and *Time point 2* (Figs. 1e, 5g, h).

In this case, finding seems to suggest that, during differentiation, the detrimental effect on neurogenesis and apoptosis caused by the high IL6 concentrations in serum from delirium patients is mediated by production of IL13. Indeed, treatment of cells with an antibody against IL13 prevented the decrease in neurogenesis (DCX, Map2) and increase in apoptosis (CC3) caused by treatment with serum from delirium patients (*Time point 1*, delirium vs delirium + IL13 antibody; for DCX, 19.1% vs 33.8%, *p* < 0.0001; for Map2, 25.1% vs 41.4%, *p* < 0.0001; for CC3, 17.3% vs 3.8%, *p* < 0.0001). Again, there were no differences between *Time point 1* and *Time point 2* (Figs. 1c, 5i–n).

### Treatment with recombinant IL12 or IL13, at concentrations previously found in supernatant of cells exposed to serum from patients with delirium, decreases cell proliferation, neurogenesis and increase apoptosis, whereas co-treatment with the JAK inhibitors, baricitinib, ruxolitinib and tofacitinib, prevents these detrimental effects

Having identified the downstream production of IL12 and IL13 in supernatant of cells exposed to serum from patients with delirium as the mechanism responsible for the reduced cell proliferation and neurogenesis and increased apoptosis, we decided to test whether (1) these same effects could be replicated by treating cells directly with IL12 and IL13 at the same concentrations measured in the supernatant following treatment with serum; and (2) if treatment with effective and commonly administered therapeutic compounds for patients with acute COVID-19, the JAK inhibitors baricitinib, ruxolitinib and tofacitinib (all 1 nM), could prevent these detrimental effects by IL12 and IL13.

For this purpose, we treated cells directly with recombinant IL12 and IL13, used at concentrations previously found in supernatant of cells exposed to serum from patients with delirium (IL12 delirium: 20 pg/ml and IL13 delirium: 25 pg/ml) or without delirium (IL12 without delirium: 3 pg/ml, IL13 without delirium: 4 pg/ml, Fig. 1f, g), with or without the JAK inhibitors.

As hypothesised, during proliferation, treatment with IL12 (20 pg/ml) was able to cause a decrease in cell proliferation (Ki67) and increase in apoptosis (CC3), when compared with IL12 (3 pg/ml) (for Ki67, 42.8% vs 71.6%, *p* < 0.0001; for CC3, 11.5% vs 5.6%, *p* < 0.0001, Supplementary Fig. 5a, b). This is similar to what previously shown upon exposure of cells to serum from patients. Interestingly, co-treatment with baricitinib, ruxolitinib or tofacitinib were able to prevent these detrimental effects (IL12 20 pg/ml vs IL12 3 pg/ml + vehicle/baricitinab/ruxolitinab/tofacitinab; for Ki67, 42.8% vs 68.4%, *p* < 0.001, vs 69%, *p* < 0.001, vs 71.4%, *p* < 0.001; for CC3, 11.5% vs 5.7%, *p* < 0.0001, vs 5%, *p* < 0.0001, vs 5.4%, *p* < 0.0001, Supplementary Fig. 5a, b).

Similarly, during differentiation, treatment with IL13 (25 pg/ml) was able to cause a decrease in neurogenesis (DCX and Map2) and increase in apoptosis (CC3), when compared with IL13 (4 pg/ml) (for DCX, 20.8% vs 31%, *p* < 0.0001; for Map2, 27.2% vs 41%, *p* < 0.0001; for CC3, 15.8% vs 7.6%, *p* < 0.0001, Supplementary Fig. 5c–e). This is, again, similar to what previously shown upon exposure of cells to serum from patients. Again, co-treatment with baricitinib, ruxolitinib or tofacitinib were able to prevent these detrimental effects (IL13 25 pg/ml vs IL13 4 pg/ml + vehicle/baricitinab/ruxolitinab/tofacitinab; for DCX, 20.8% vs 30.4%, *p* < 0.001, vs 29%, *p* < 0.001, vs 30%, *p* < 0.001; for Map2, 27.2% vs 39.1%, *p* < 0.001, vs 39.7%, *p* < 0.001, vs 39.7%, *p* < 0.001; for CC3, 15.8% vs 7.4%, *p* < 0.0001, vs 8.3%, *p* < 0.0001, vs 8.1%, *p* < 0.0001, Supplementary Fig. 5c–e).

## Discussion

To our knowledge, this is the first study to identify the high IL6 levels in the serum of COVID-19 hospitalised patients with neurological symptoms (delirium) as the molecular mechanism leading to disruption in neurogenic processes, measured in vitro using a unique human model of hippocampal precursors. Interestingly, IL-6-induced increased production by these hippocampal cells of IL12 (during proliferation) and IL13 (during differentiation) is the molecular mechanisms downstream to IL6, suggesting that brain production of cytokines in response to peripheral inflammation is important in neurological symptoms induced by COVID-19 infection. Indeed, treatment with an antibody against IL12 or IL13 prevented the effect of serum from delirium patients on cell proliferation, cell differentiation and apoptosis. Of note, treatment with the therapeutic compounds commonly administered to acute COVID-19 patients (the JAK inhibitors baricitinib, ruxolitinib and tofacitinib) were able to restore normal cell viability, proliferation and neurogenesis by targeting the effects of IL12 and IL13. The novelty of our study resides in the use of a well-established but, at the same time, unique model of human hippocampal neurogenesis [25, 30–34], and a validated “blood-brain-axis” experimental assay [28] using serum samples from COVID-19 patients.

While there is evidence showing that patients with COVID-19 present with neurological symptoms both during and after the disease (the latter phenomenon is the so-called “long COVID”) [7, 8, 41], the brain mechanisms through which this occur have not been identified yet. So far there is some indication that the SARS-CoV-2 can affect transcriptionally brain cells from the parenchymal and choroid plexus, and is able to reduce hippocampal neurogenesis in animal and human models of COVID-19 [42–44]. Similarly, there is evidence for an association between neuroinflammation and neurological symptoms during the acute and mild phase of COVID-19 [44–46]. Our study identifies IL6 and IL6-induced IL12 and IL13 as the putative mechanisms mediating these effects. Specifically, we found a 6-fold increase in the concentrations of IL6 in serum samples of patients with delirium (229.9 ± 105.2 pg/ml), when compared with patients without delirium (32.5 ± 9.5 pg/ml). In line with our findings, higher levels of IL6 have been reported also by other studies in the periphery and in the cerebrospinal fluid (CSF) of COVID-19 patients [12, 13, 21, 47, 48], as well as in children with acute encephalitis-like syndrome during infection with coronavirus-OC43 [49]. Overall, these findings identify IL6 an important therapeutic target not only for COVID-19 symptoms in general, but also for those neurological manifestations which are commonly observed as a consequence of the disease. Interestingly, this result is consistent with our recent study showing that exposing the same cells to concentrations of IL6 ranging from 50 to 5000 pg/ml dramatically decrease neurogenesis and increase apoptosis [22]. Likewise, previous findings generated in other laboratories also demonstrated that exposure to concentrations of IL6 similar to those detected in the present study is associated with a significant reduction in neurogenesis and increase in apoptosis, both in vitro and in vivo [50–52].

Of note, treatment with serum from delirium patients induced our cells to produce two well-known inflammatory cytokines, IL12 and IL13, in the supernatant; these cytokines were in very low quantities in serum, indicating a direct production by cells. The causal role of IL6 and IL6-induced IL12 and IL13 in mediating the effect of serum from delirium patients on the neurogenic outcomes is confirmed by the use of an antibody against these cytokines in our experiments. Interestingly, IL12 was produced only during the proliferation stage, whereas IL13 only during the differentiation stage, but always as a consequence of IL6 stimulation. Interestingly, while both IL12 and IL13 are expressed in the brain [53, 54], IL12 is primarily known to regulate cell proliferation [53], whereas IL13 is mainly involved in differentiation-related processes [54]. Indeed, IL12 reduces cell proliferation and migration in several neuronal cultures via activation of distinct pro-inflammatory signalling pathways, including janus-activated kinase and signal transducers and activators of transcription (JAK-STAT) pathway [55, 56], which we have also previously identified to be involved in the regulation of neuronal apoptosis upon exposure of hippocampal cell to IFN-α treatment [24]. Accordingly, previous evidence has reported an increase in the production of IL12 and IL13 in the CSF of COVID-19 patients with neurological manifestations [57, 58]. Similarly, in mice, studies have shown that increased cerebral expression of IL12 is able to induce neurological symptoms, and that these symptoms are worsen by an infectious disease [53]. Likewise, in humans, IL13 expression has shown to be associated with the development of neurological disorders, and with the experience of more severe neuropsychiatric symptoms. In particular, individuals with high IL13 expression are more likely to develop neurological diseases, including Parkinson’s [59], whereas in those individuals who already suffer from a major depressive disorder, higher IL13 levels are associated with more severe depression and a higher number of suicide attempts [60].

Our study identifies multiple pharmacological approaches that could be beneficial for treatment of neurological symptoms in the. context of COVID-19 infection, and thus potentially in other conditions associated with high levels of IL6. First, IL6 itself can be considered a valuable therapeutic target in treating COVID-19 patients. Indeed, treatment with IL6 receptor antagonists, such as tocilizumab or sarilumab, have so far shown a relatively good level of efficacy in reducing COVID-19 mortality rates, although these compounds have a high molecular weight, making them unable to act centrally [61]. Second, there are several IL12 inhibitors available, such as ustekinumab and briakinumab, which have already been tested for their efficacy and tolerability in clinical context [62]. However, these compounds are not exclusively targeting IL12, but rather the p40 subunit of both IL12 and IL23, and have a relatively high molecular weight, making them less likely to exert their properties centrally [63]. The situation is similar for IL-13: although a selective inhibitor, lebrikizumab, has already been developed and tested in a phase 2b trial, this compound also has a very high molecular weight, making it unsuitable for treatment of brain inflammatory neuropathologies [64]. Finally, we have shown that the JAK inhibitors, baricitinib, ruxolitinib and tofacitinib, prevent the reduction in cell proliferation and neurogenesis, and the increase in apoptosis, caused by treatment with recombinant IL12 or IL13. Recently randomized, placebo-controlled trials of these have shown these compounds to be among the most effective treatment interventions for acute COVID-19 patients, especially for those with a high level of inflammation [65–67]. Moreover, these JAK inhibitors have low molecular weight (<400 Da) making them suitable to act centrally, and potentially on mechanisms linked to the neuronal production of IL12 and IL13 that we describe. All together, we propose that baseline stratification of patients based on their level of inflammatory biomarkers, including IL6, IL12 and IL13, or the presence of neurological symptoms, may represent the best therapeutic approach for those individuals experiencing severe COVID-19 infection.

We acknowledge several limitations associated with our study, including the use of an in vitro system of immortalized cell line. However, while theoretically this system may differ from the scenario of an adult in vivo environment and the adult neurogenic niche, in particular because of the absence of microglia cells and the foetal status of the immortalized cells, we have extensive confirmation from previous studies that our results are relevant to the human and animal brains. Indeed, over the years we have been able to replicate all our results obtained in this in vitro model in both the animal brain and the human blood, including changes in neurogenesis by cortisol, antidepressants, IL1β, IFN-α, and serum samples from depressed patients, and changes in stress-, antidepressants- and inflammation-regulated mRNA gene expression in both the hippocampal mRNA of animal models of depression and the whole blood mRNA of depressed patients [22, 25, 28, 30–34, 40, 68]. Nevertheless, in future studies, we aim to expand this cellular model using both neurons and microglia co-cultures, in order to create a more comprehensive and realistic experimental scenario. Also, our study does not investigate the direct effects of COVID-19 on these cells. However, considering that there is evidence showing that the virus has difficulty crossing the BBB in humans, and that its central effect is instead mediated by the activation of peripheral mechanisms [44, 69, 70], we believe that our experimental approach, exposing brain cells to serum from COVID-19 infected patients rather than to the virus itself, provides mechanistic understanding on how COVID-19 indirectly regulates brain function. Finally, the number of samples used in this study, although matched for age and sex, was also relatively small, 18 patients per group. Therefore, the results presented here should be replicated in a bigger cohort of COVID-19 patients with and without delirium, including patients with long COVID, who are also known to experience persistent neurological manifestations [41]. However, while the sample size is small, this is the first study demonstrating for the first time that it is the elevated peripheral concentration of IL-6 (rather than of other pro-inflammatory cytokines) that clinically affects cognition and mechanistically affects neurogenesis in the same COVID-19 patients with delirium. In addition, the identification of the IL12 and IL13 as downstream mediators is a completely novel mechanism for the IL-6-induced reduction in neurogenesis, and the novel experiments using JAK inhibitors performed in the present study offer additional mechanistic insights into these pathways.

In summary, our in vitro study demonstrates that that serum from COVID-19 patients with delirium can detrimentally affect human hippocampal neurogenesis, and that this effect is mediated by IL6-induced production of the downstream inflammatory cytokines IL12 and IL13, which are ultimately responsible for the negative cellular outcomes. Understanding the mechanisms through which neurogenesis is altered in COVID-19 patients with neurological symptoms will ultimately contribute to the development of novel interventional and preventative strategies for this particular sub-group of patients, and potentially for patients with cognitive and neurological symptoms in the context of inflammation.

## Supplementary information

Supplementary Figure 1

Supplementary Figure 2

Supplementary Figure 3

Supplementary Figure 4

Supplementary Figure 5

Supplementary Legends
