# Supplementary figures and images for: Neurogenesis is disrupted in human hippocampal progenitor cells upon exposure to serum samples from hospitalized COVID-19 patients with neurological symptoms
Source: Mol Psychiatry. 2022 Oct 5;27(12):5049–61. doi: 10.1038/s41380-022-01741-1 (PMC9763123; doi:10.1038/s41380-022-01741-1)

**a)**

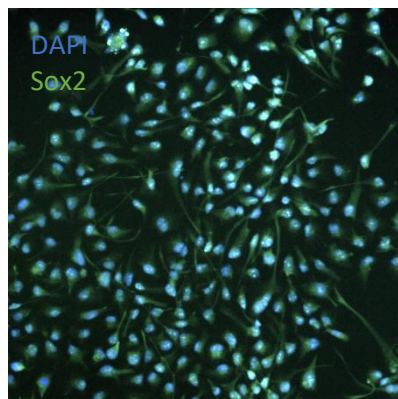

**b)**

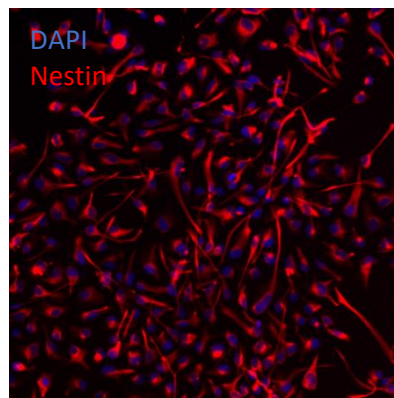

**c)**

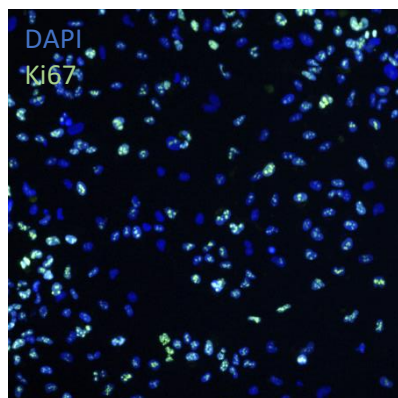

**d)**

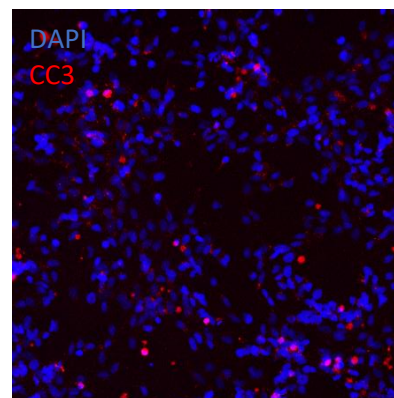

**e)**

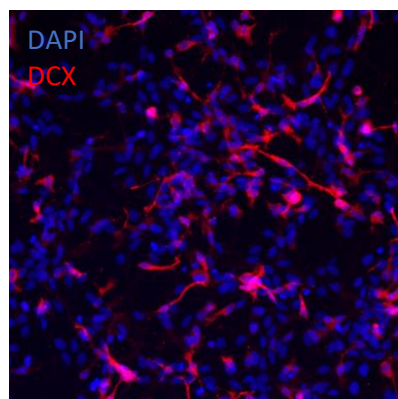

**f)**

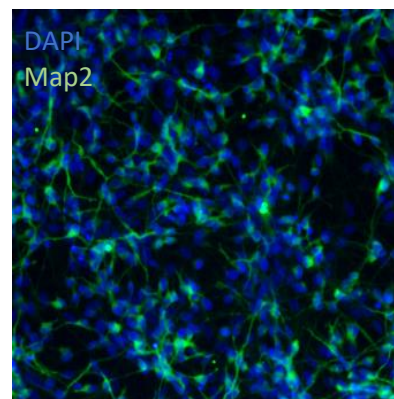

**g)**

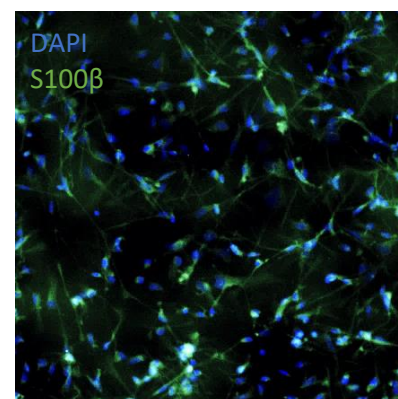

**h)**

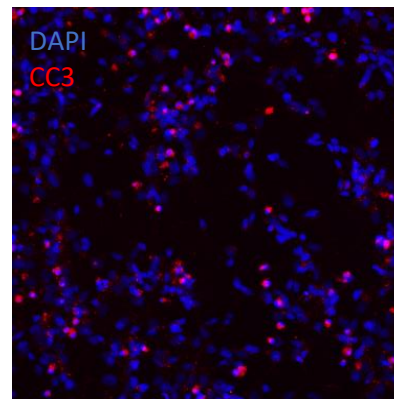

i)

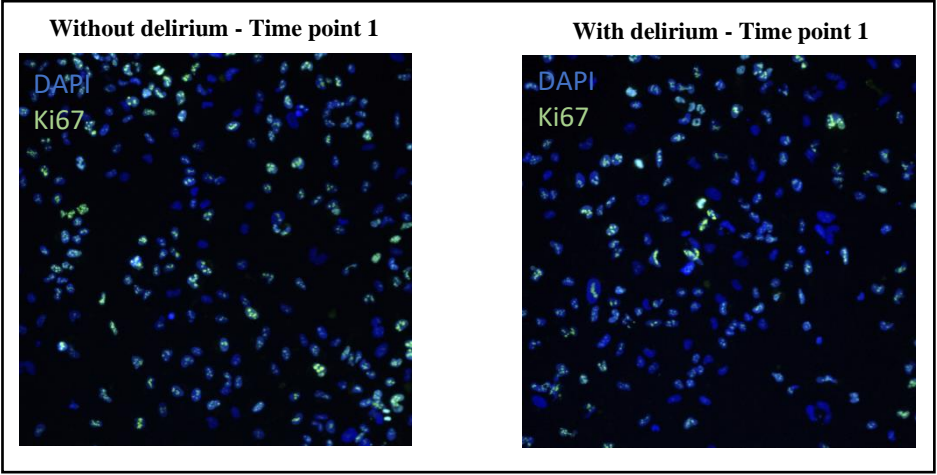

j)

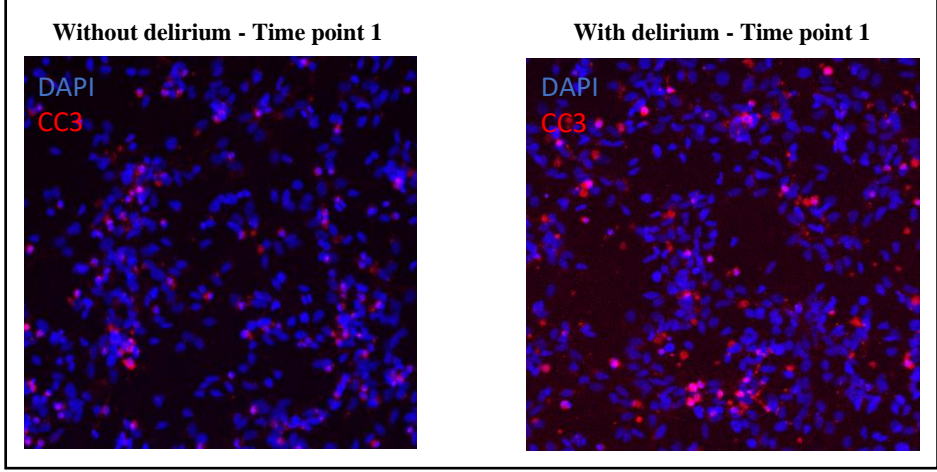

k)

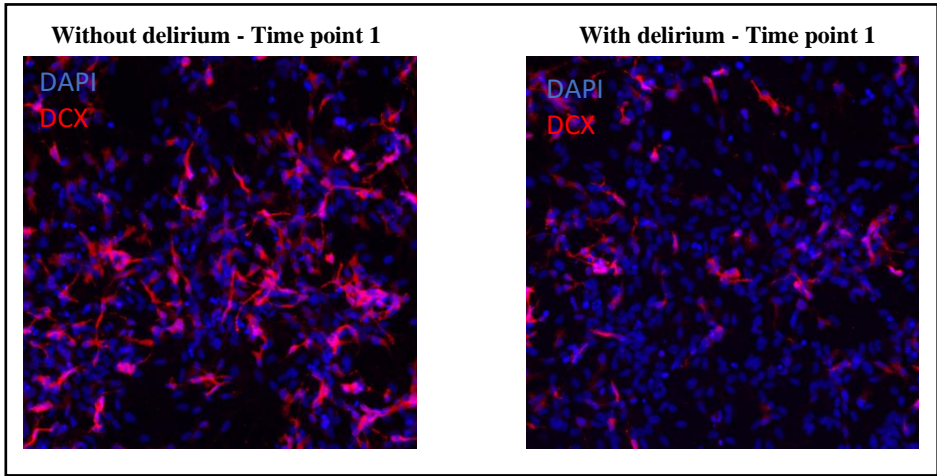

l)

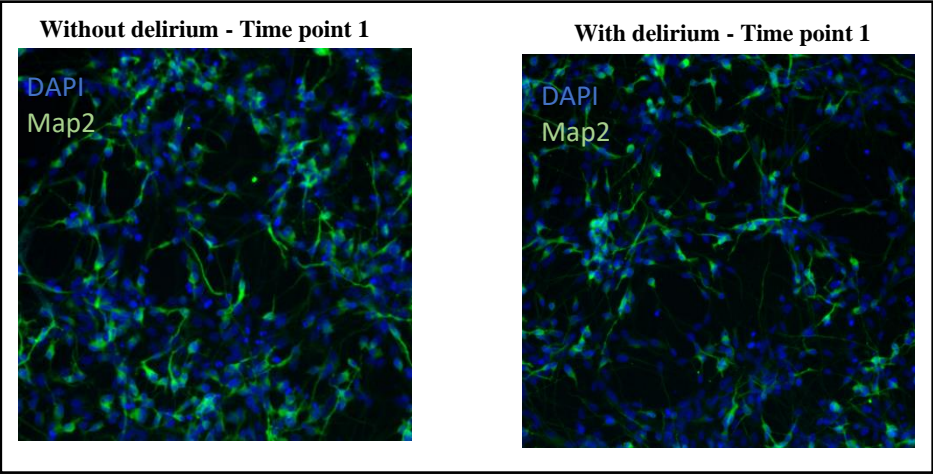

m)

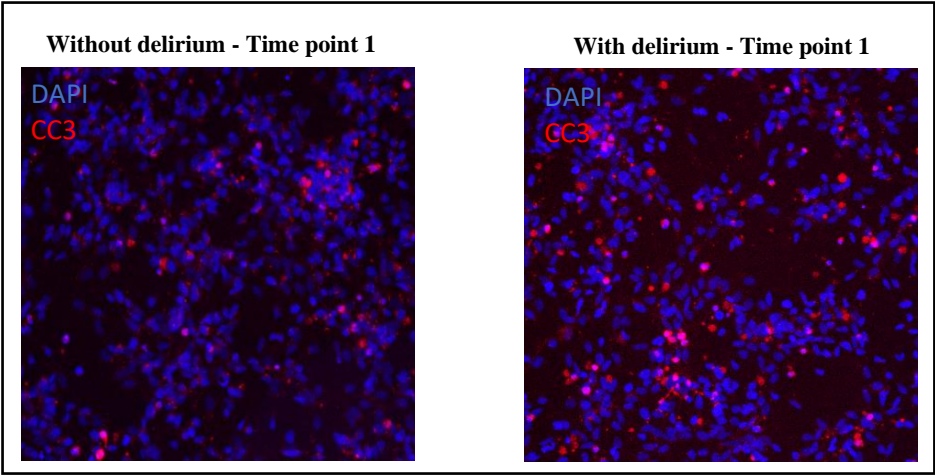

Supplement: Supplementary file 1 — Supplementary Figure 1 [file 41380_2022_1741_MOESM1_ESM.pdf]

a)

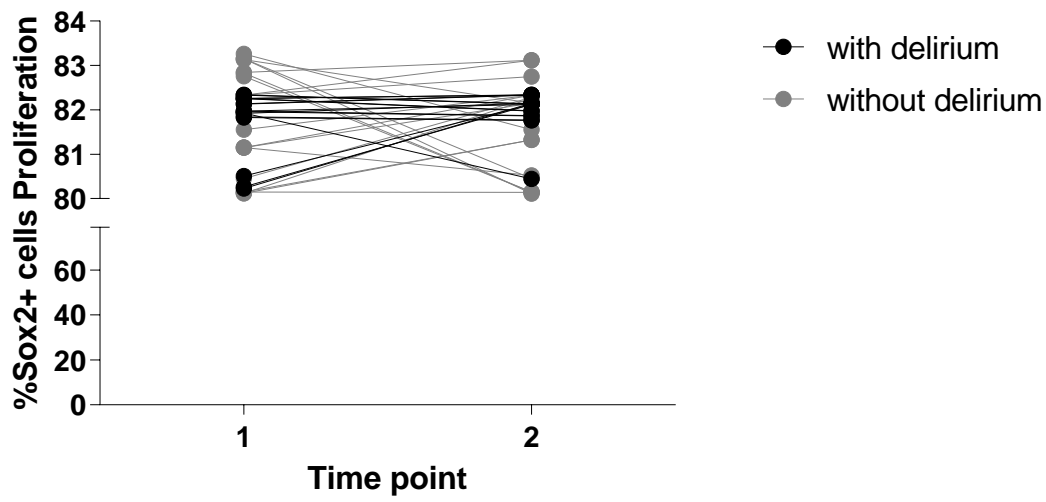

b)

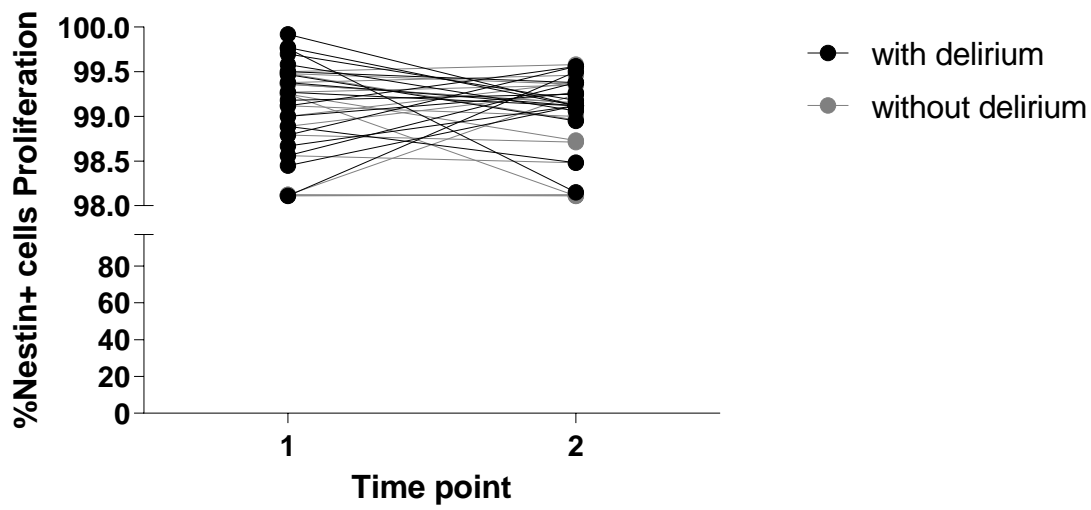

c)

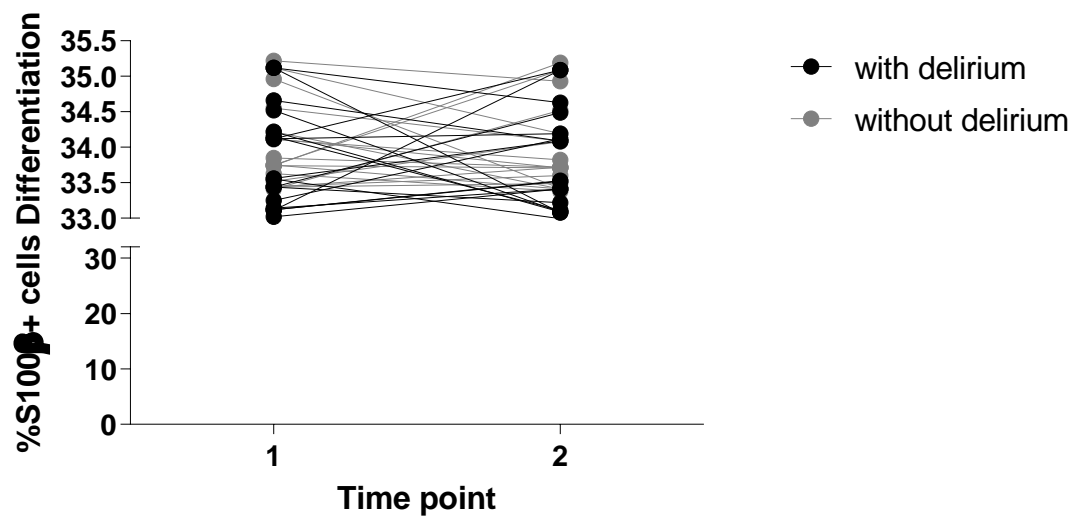

Supplement: Supplementary file 2 — Supplementary Figure 2 [file 41380_2022_1741_MOESM2_ESM.pdf]

a)

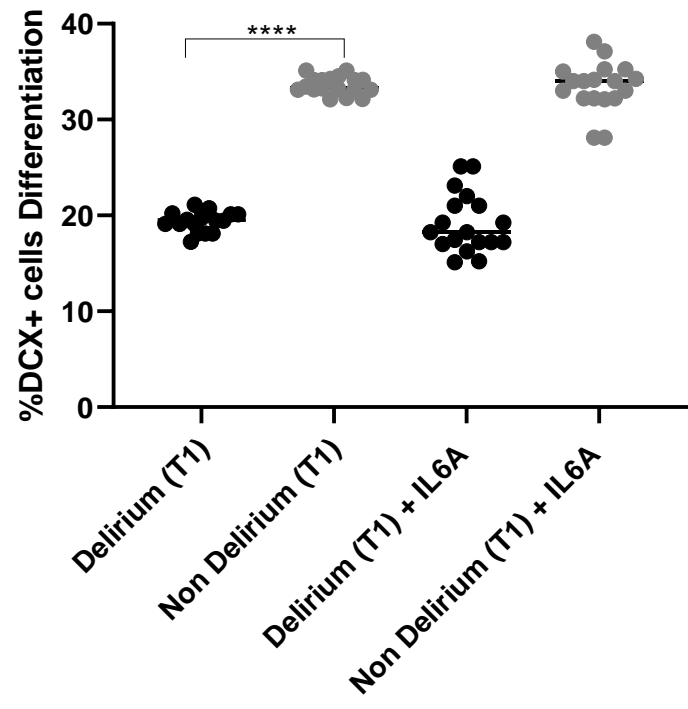

b)

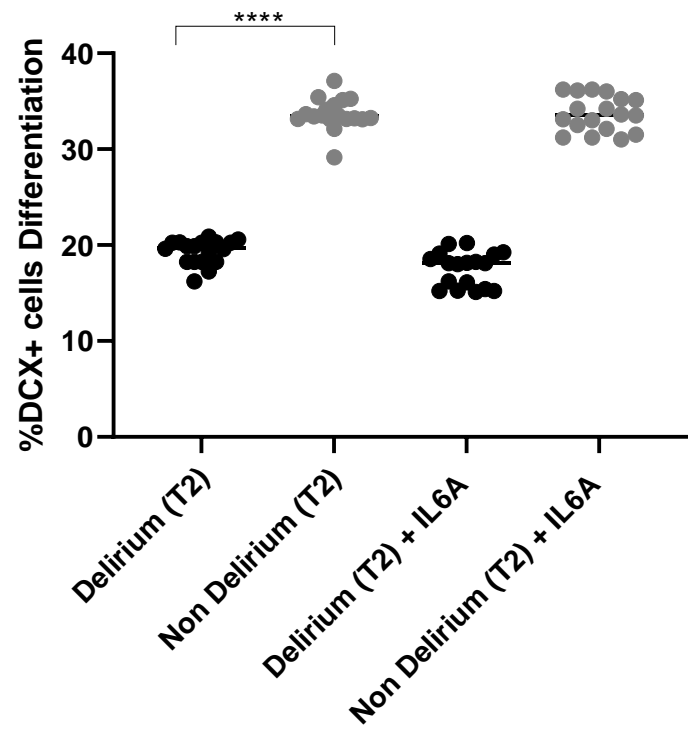

c)

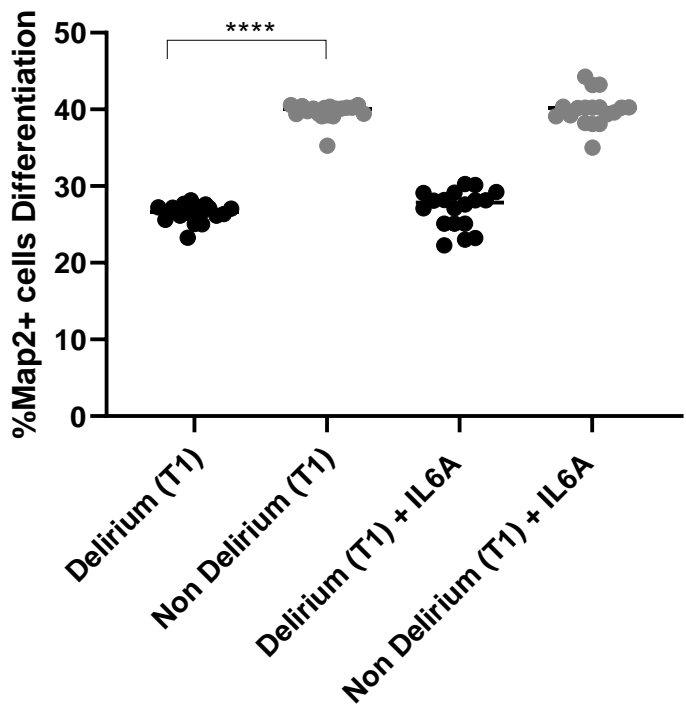

d)

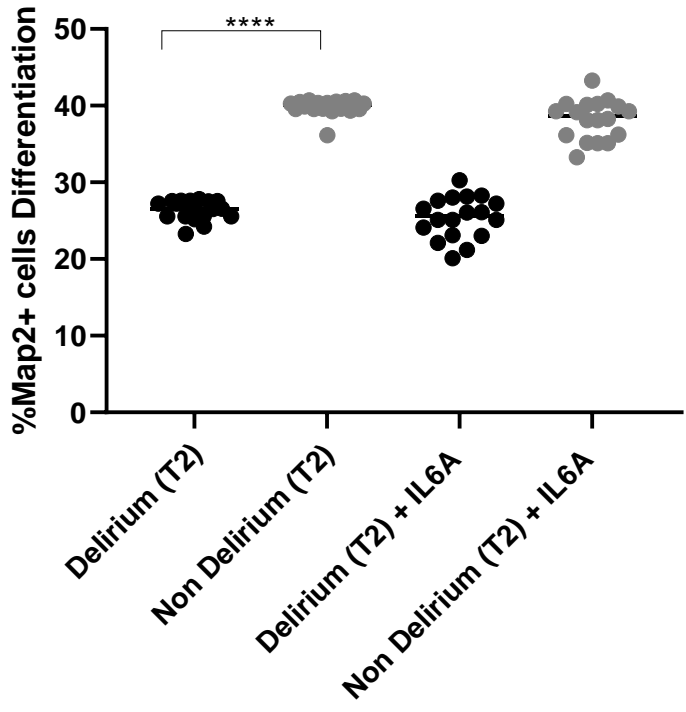

e)

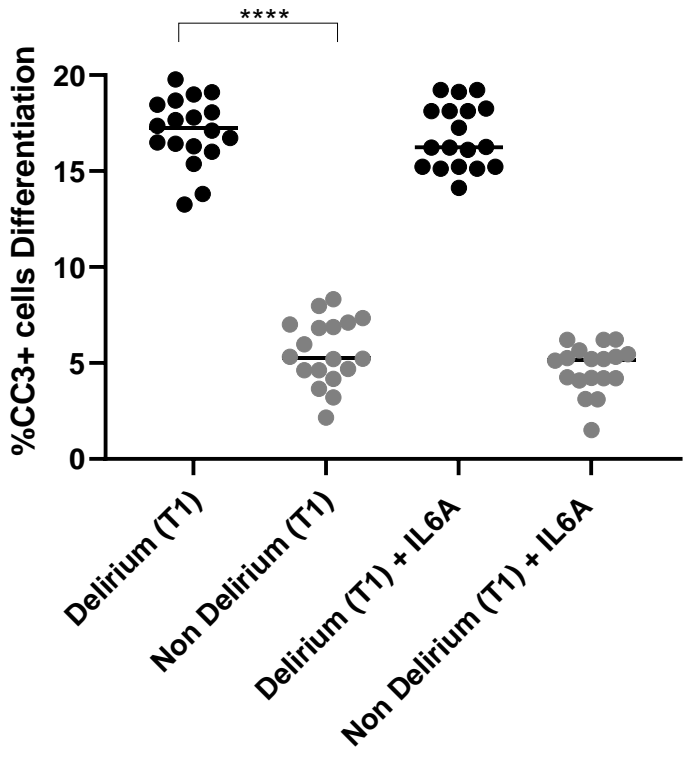

f)

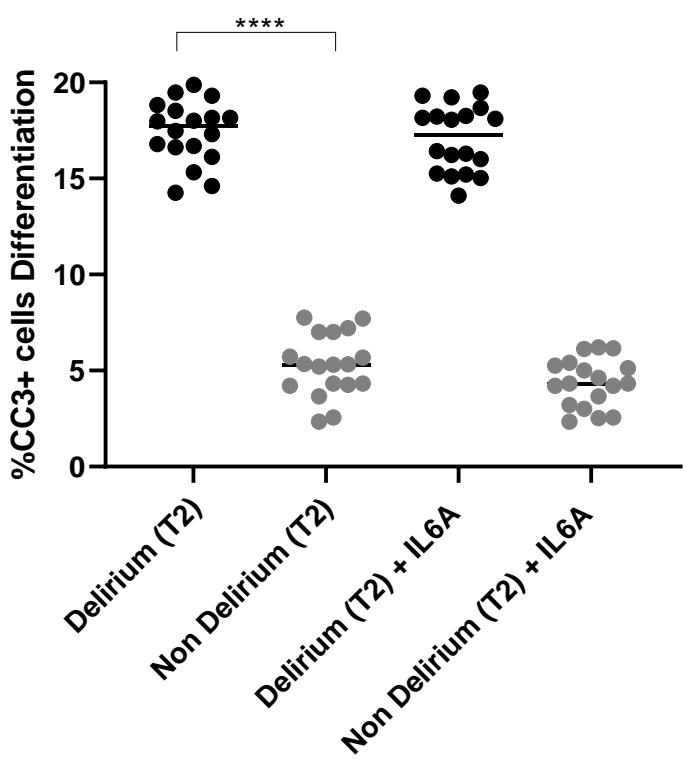

g)

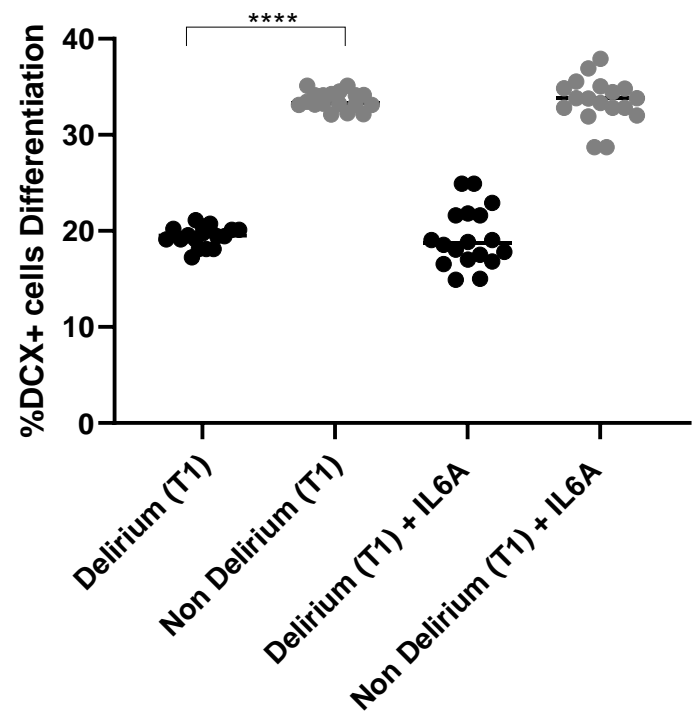

h)

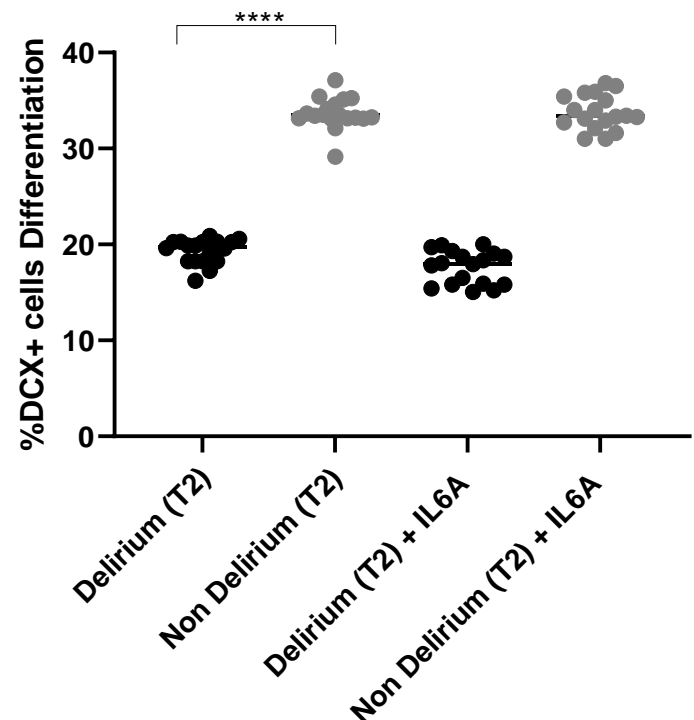

i)

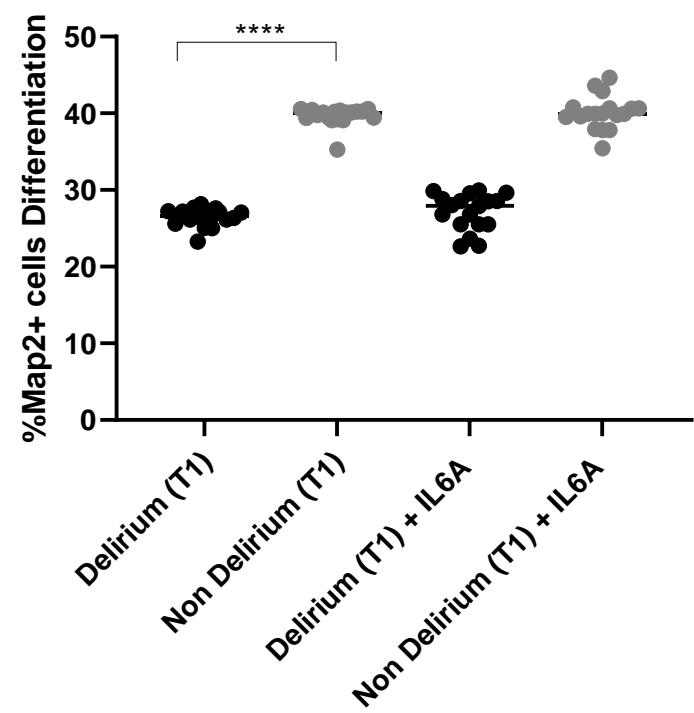

j)

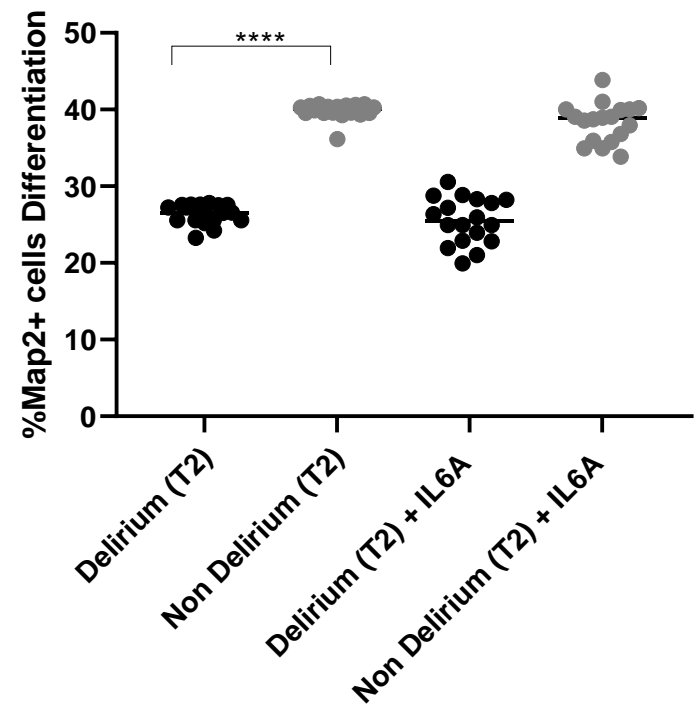

k)

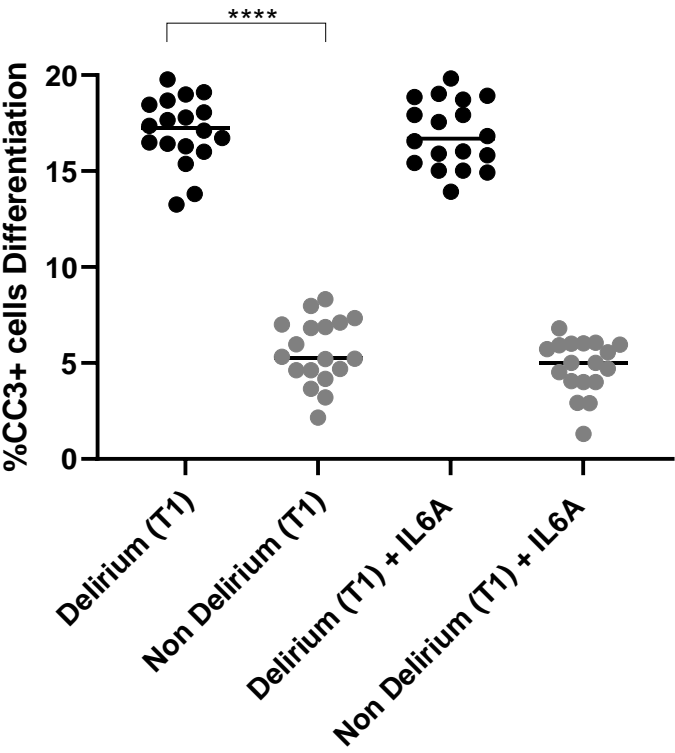

l)

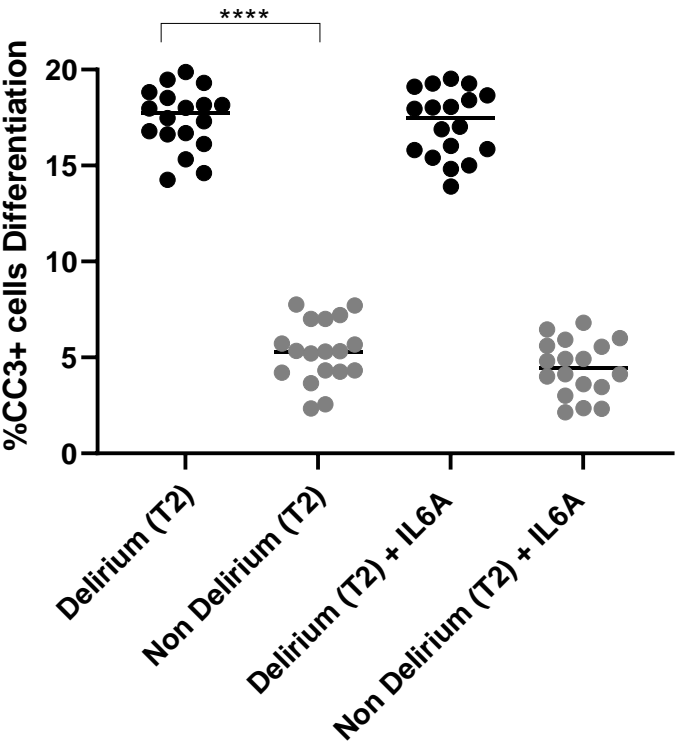

Supplement: Supplementary file 4 — Supplementary Figure 4 [file 41380_2022_1741_MOESM4_ESM.pdf]

a)

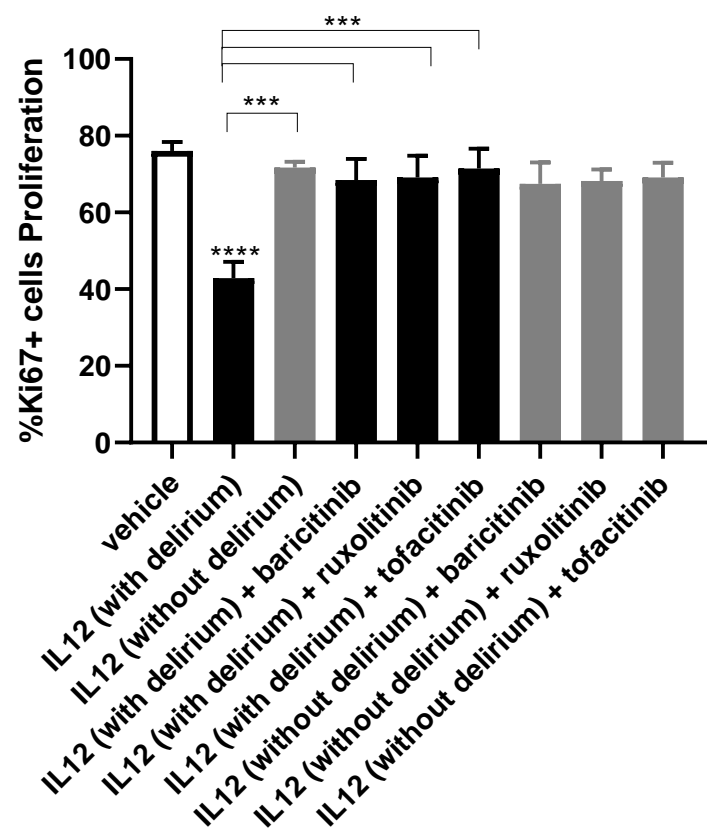

b)

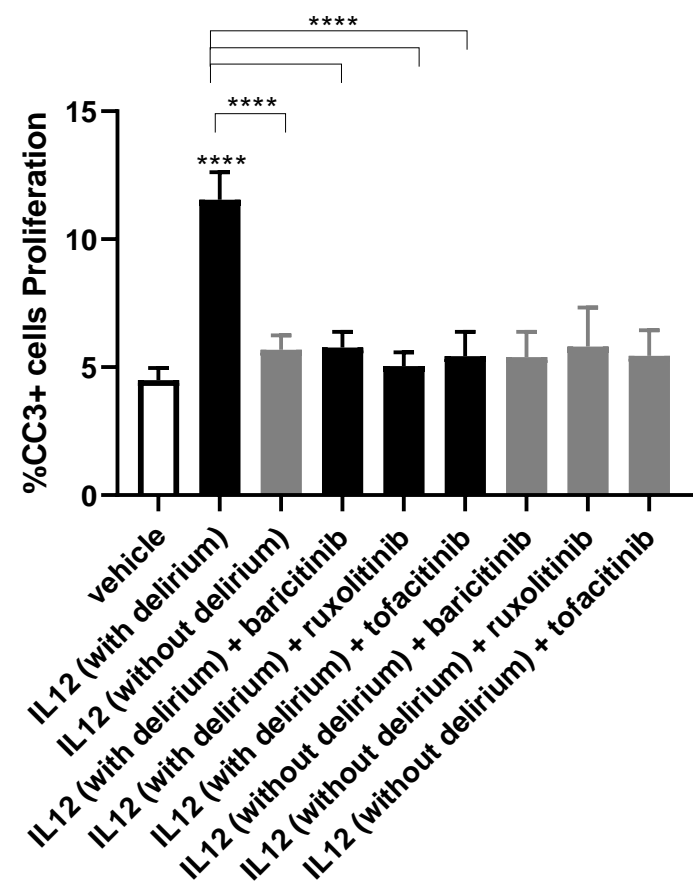

c)

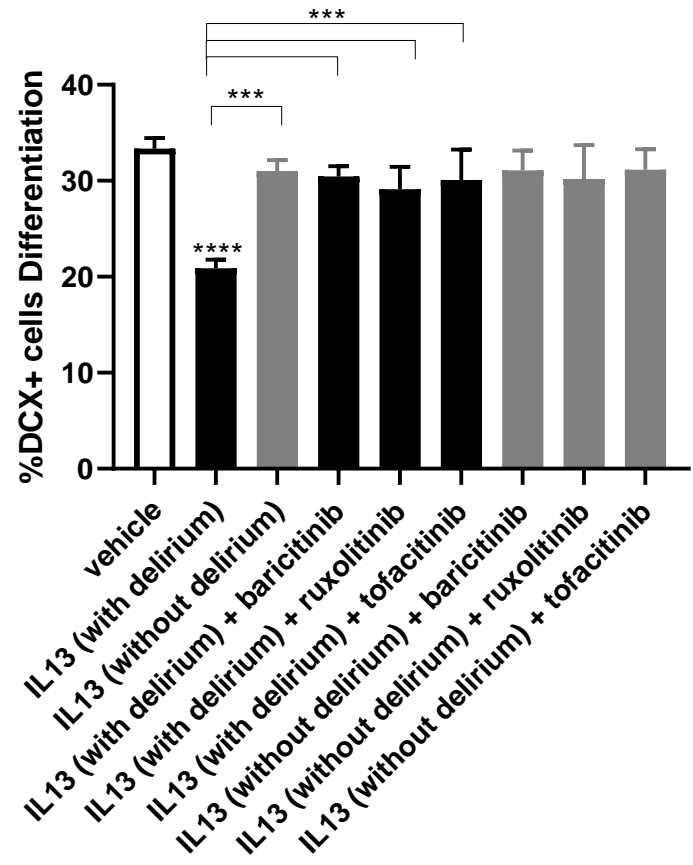

d)

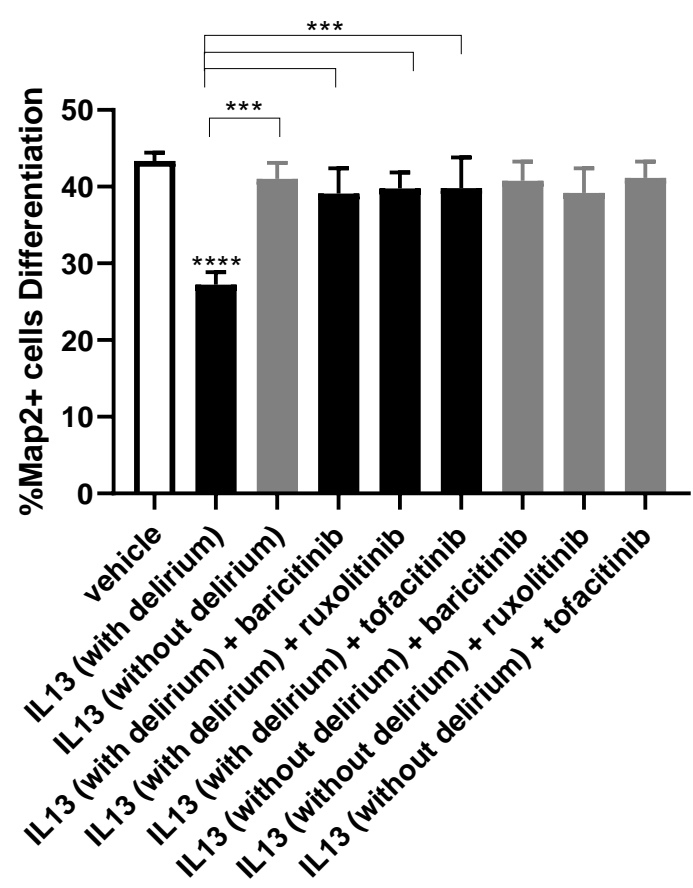

e)

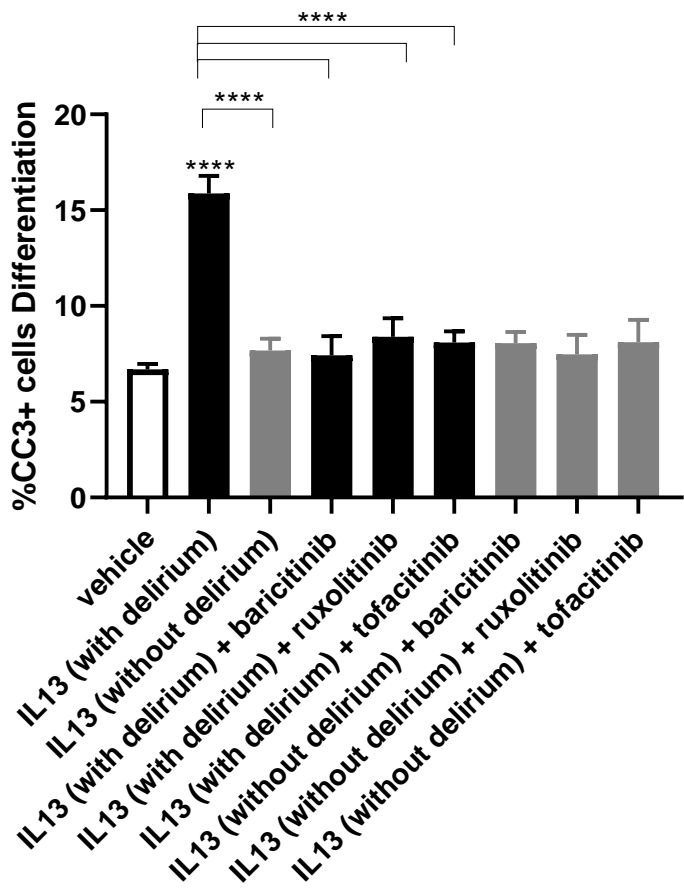

Supplement: Supplementary file 5 — Supplementary Figure 5 [file 41380_2022_1741_MOESM5_ESM.pdf]
