# Supplementary material for: Neurogenesis is disrupted in human hippocampal progenitor cells upon exposure to serum samples from hospitalized COVID-19 patients with neurological symptoms
Source: Mol Psychiatry. 2022 Oct 5;27(12):5049–61. doi: 10.1038/s41380-022-01741-1 (PMC9763123; doi:10.1038/s41380-022-01741-1)
Supplement: Supplementary file 3 — Supplementary Figure 3 [file 41380_2022_1741_MOESM3_ESM.pdf]

a)

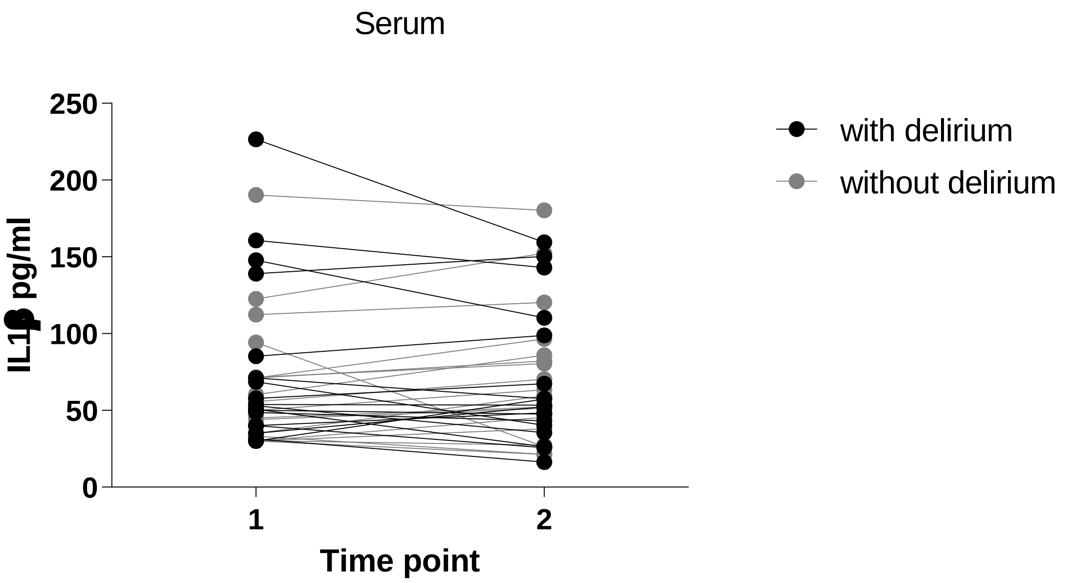
Serum
250
200
150
100
50
0
IL1β pg/ml
1
2
Time point
with delirium
without delirium

b)

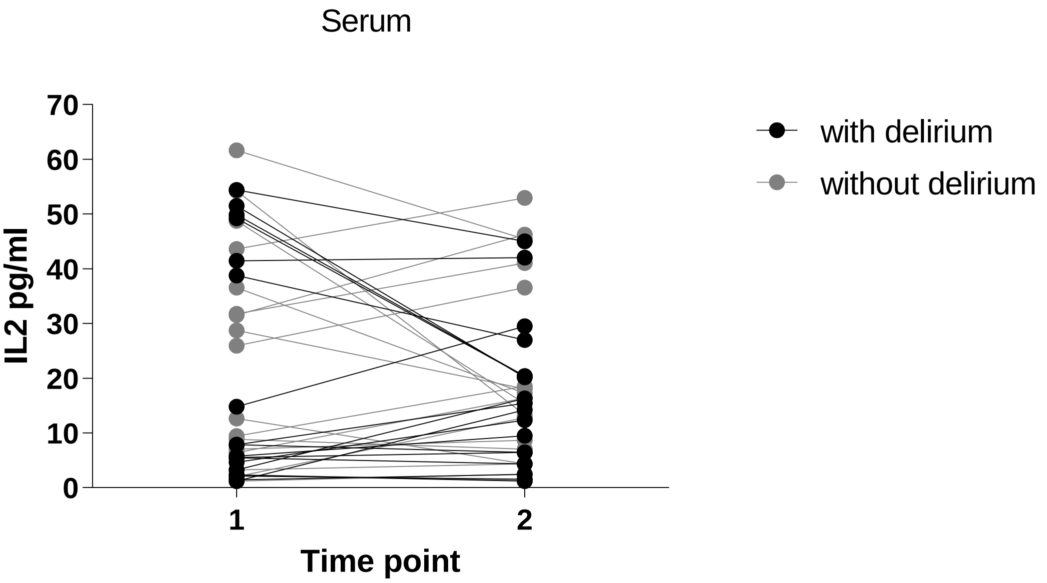
Serum
70
60
50
40
30
20
10
0
IL2 pg/ml
1
2
Time point
with delirium
without delirium

c)

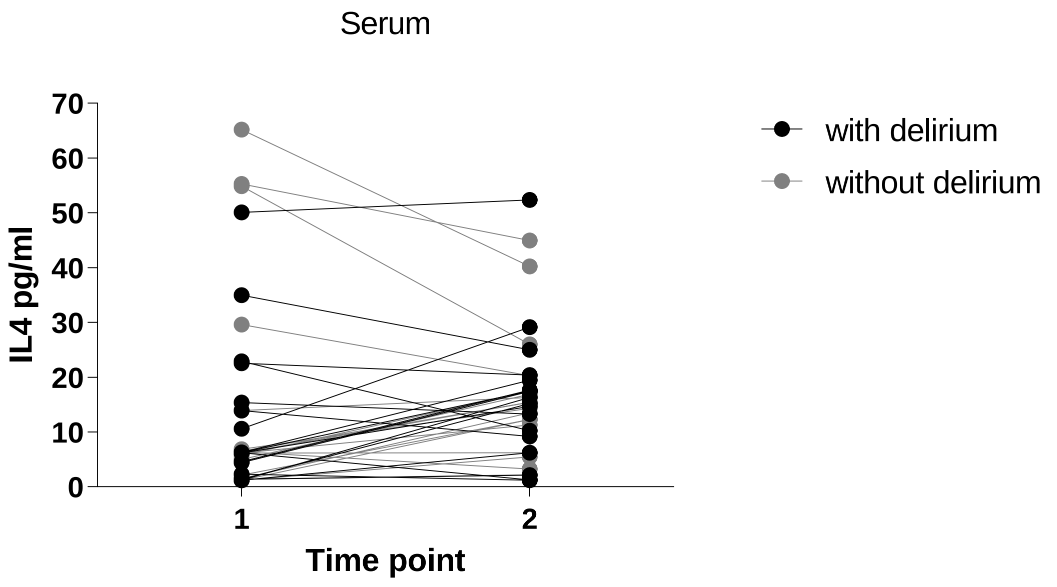

Serum
IL4 pg/ml
70
60
50
40
30
20
10
0
1
2
Time point
with delirium
without delirium

d)

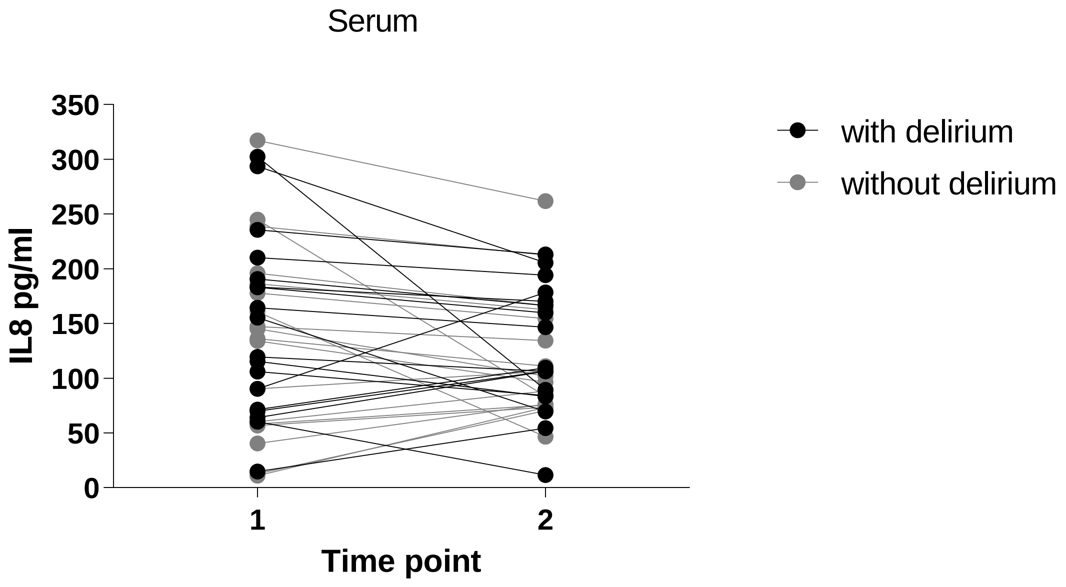

Serum
IL8 pg/ml
350
300
250
200
150
100
50
0
1
2
Time point
with delirium
without delirium

e)

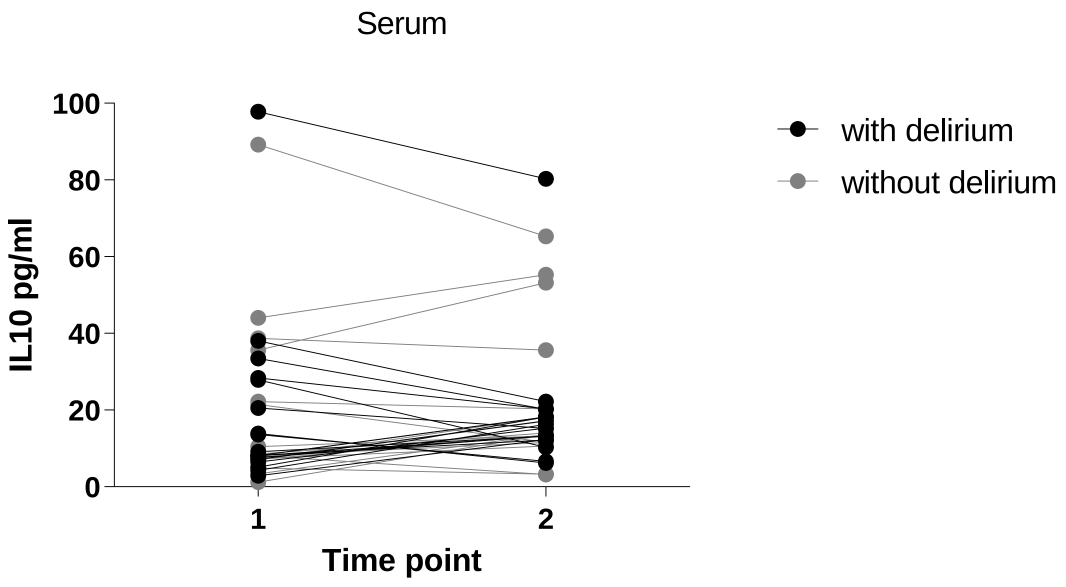

Serum
100
80
60
40
20
0
IL10 pg/ml
1
2
Time point
with delirium
without delirium

f)

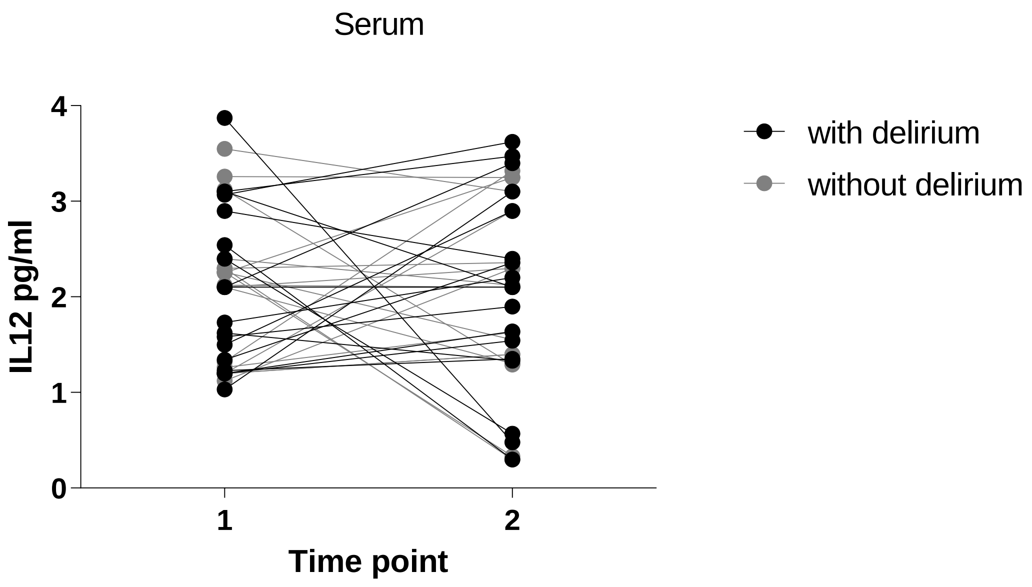

Serum
4
3
2
1
0
IL12 pg/ml
1
2
Time point
with delirium
without delirium

g)

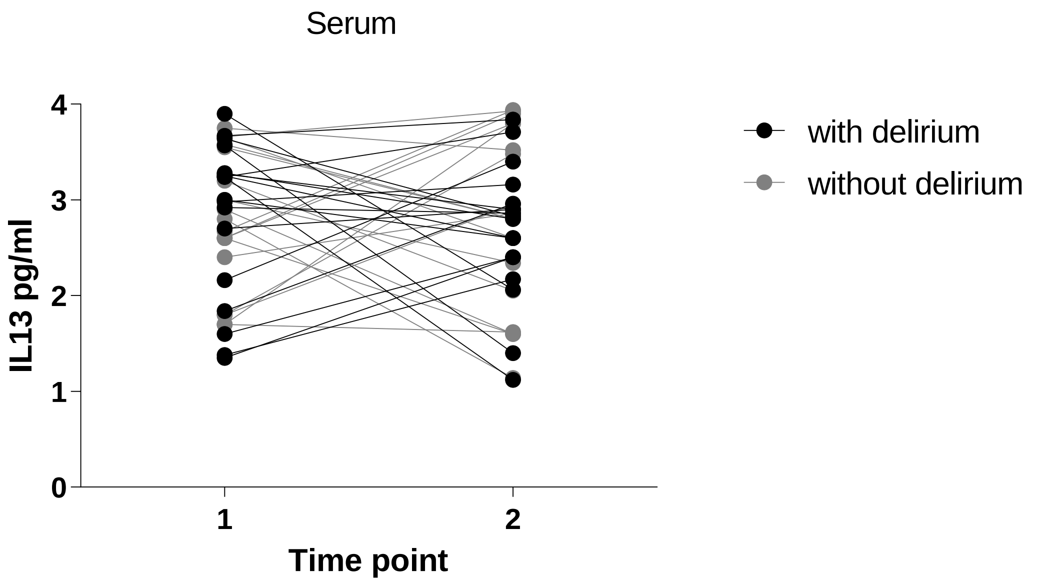
Serum
IL13 pg/ml
0
1
2
3
4
1
2
Time point
with delirium
without delirium

h)

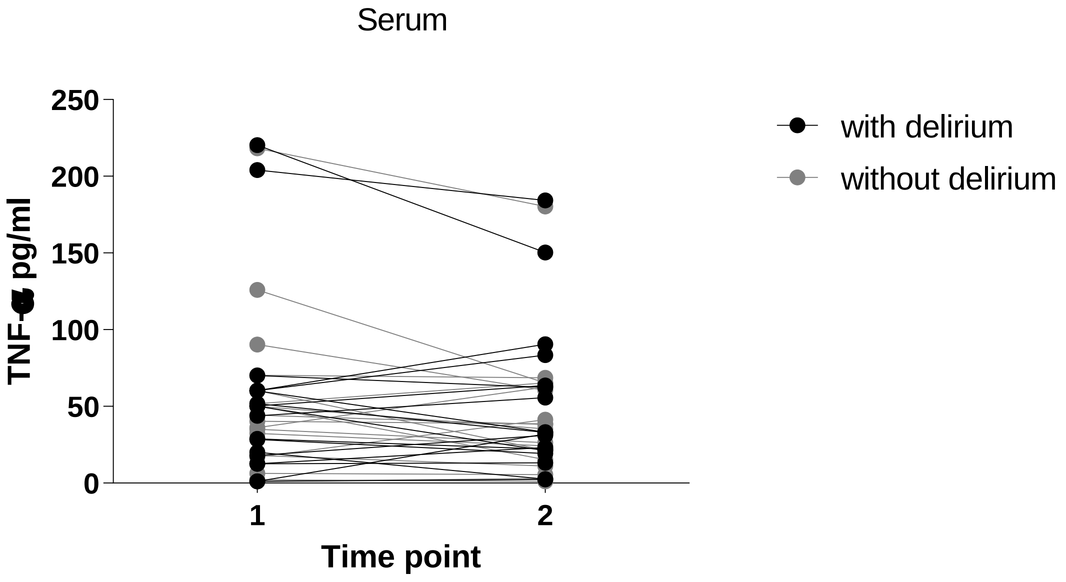
Serum
TNF-α pg/ml
0
50
100
150
200
250
1
2
Time point
with delirium
without delirium

i)

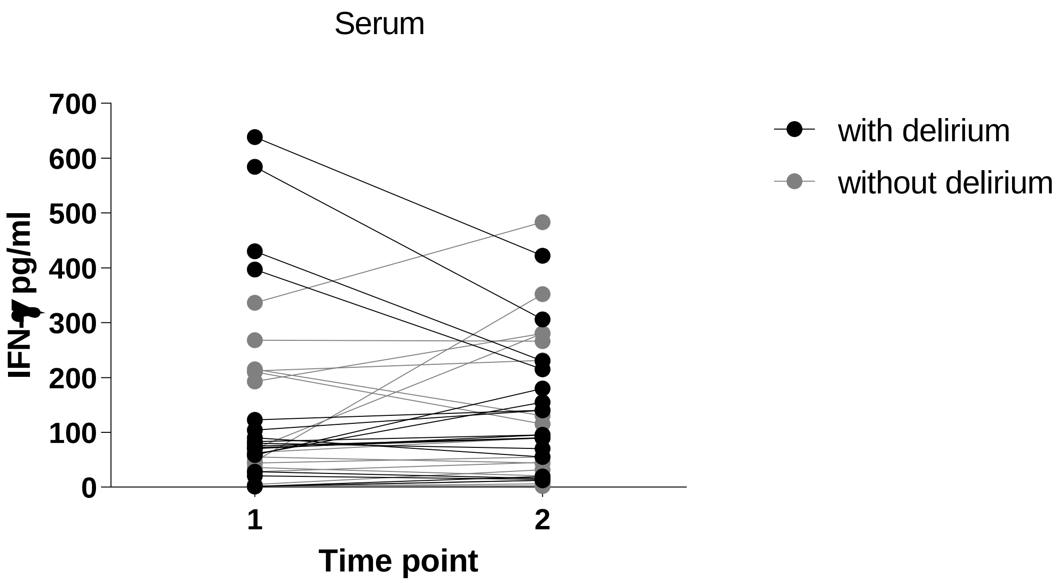
Serum
700
600
500
400
300
200
100
0
IFN-γ pg/ml
1
2
Time point
with delirium
without delirium

j)

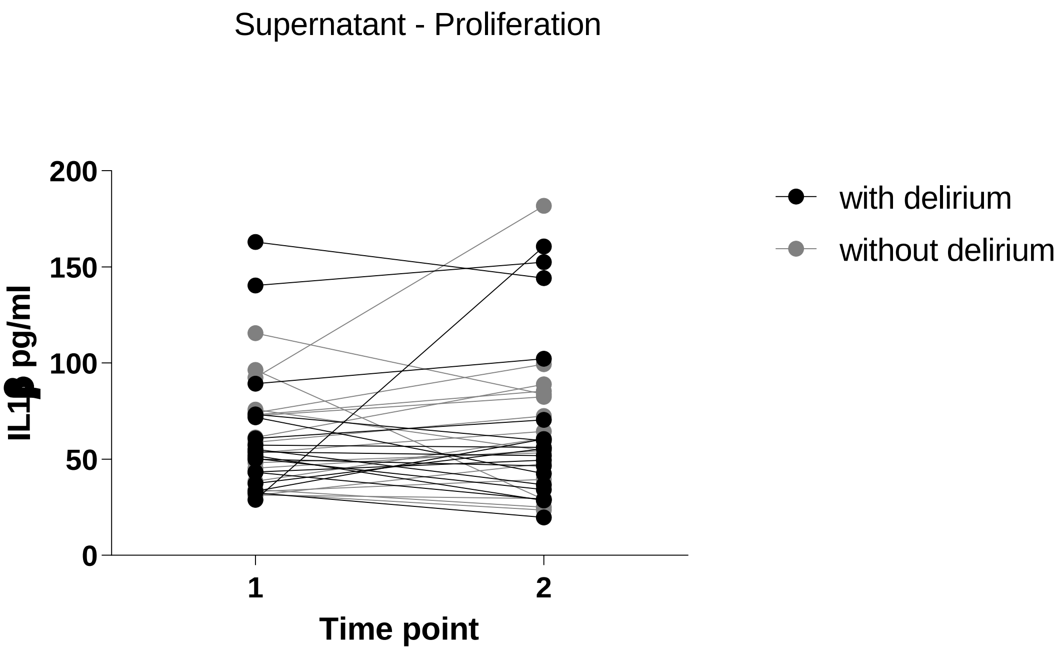
Supernatant - Proliferation
200
150
100
50
0
IL1β pg/ml
1
2
Time point
with delirium
without delirium

k)

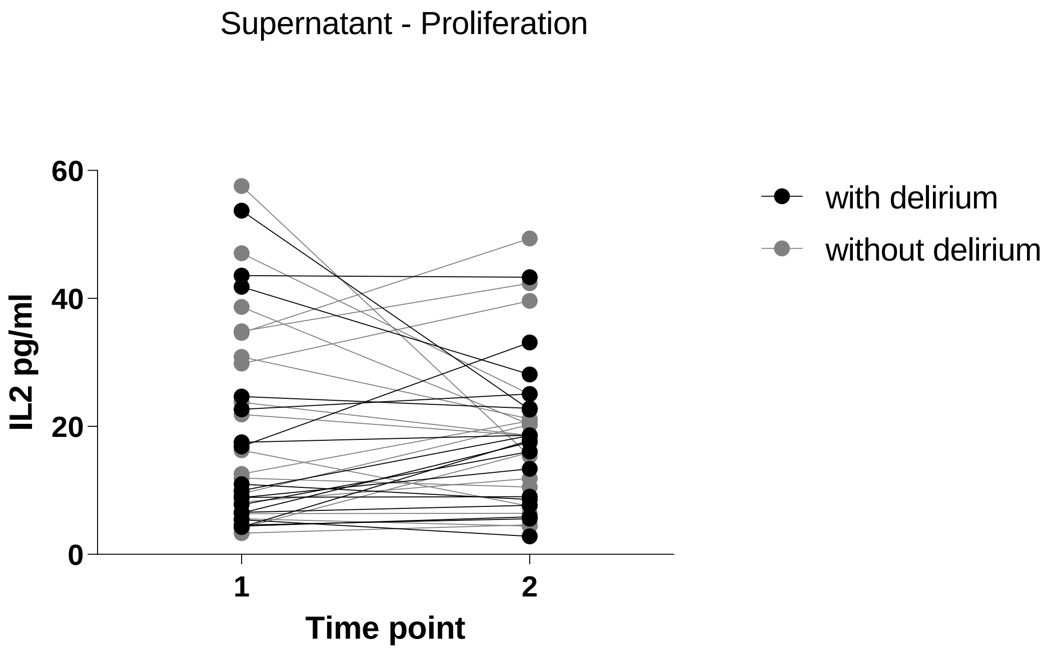

Supernatant - Proliferation
60
40
20
0
IL2 pg/ml
1
2
Time point
with delirium
without delirium

l)

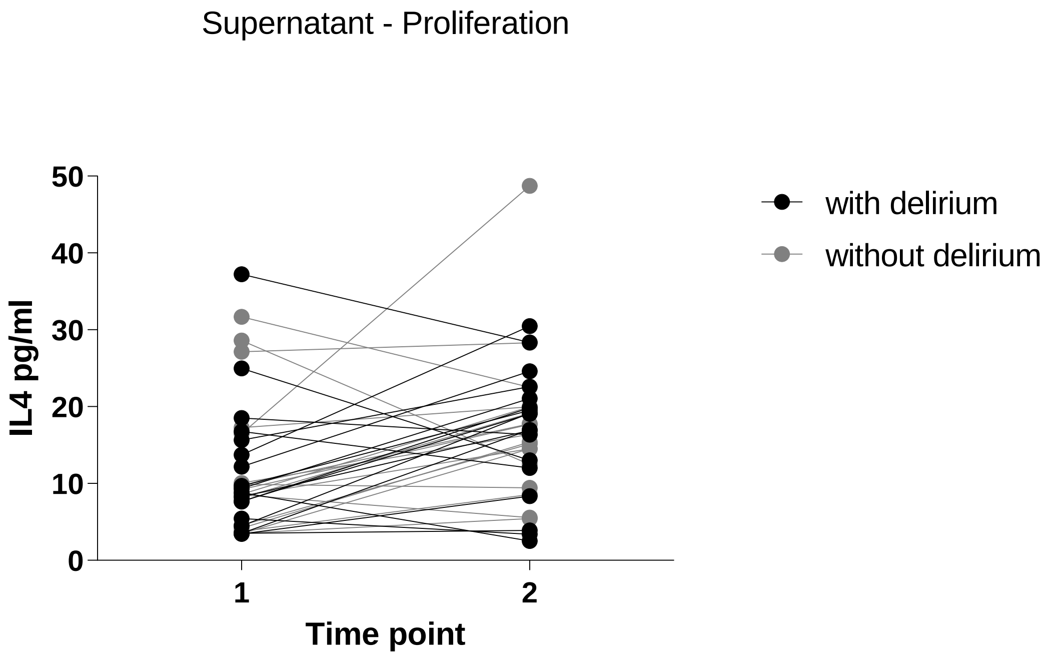

Supernatant - Proliferation
50
40
30
20
10
0
IL4 pg/ml
1
2
Time point
with delirium
without delirium

m)

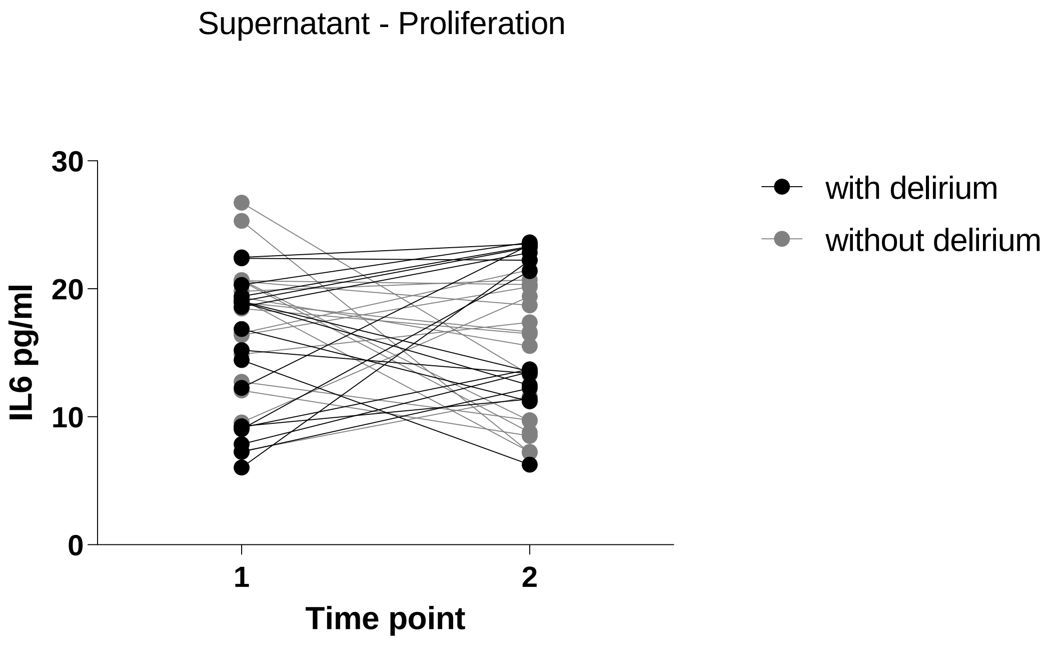
Supernatant - Proliferation
IL6 pg/ml
30
20
10
0
1
2
Time point
with delirium
without delirium

n)

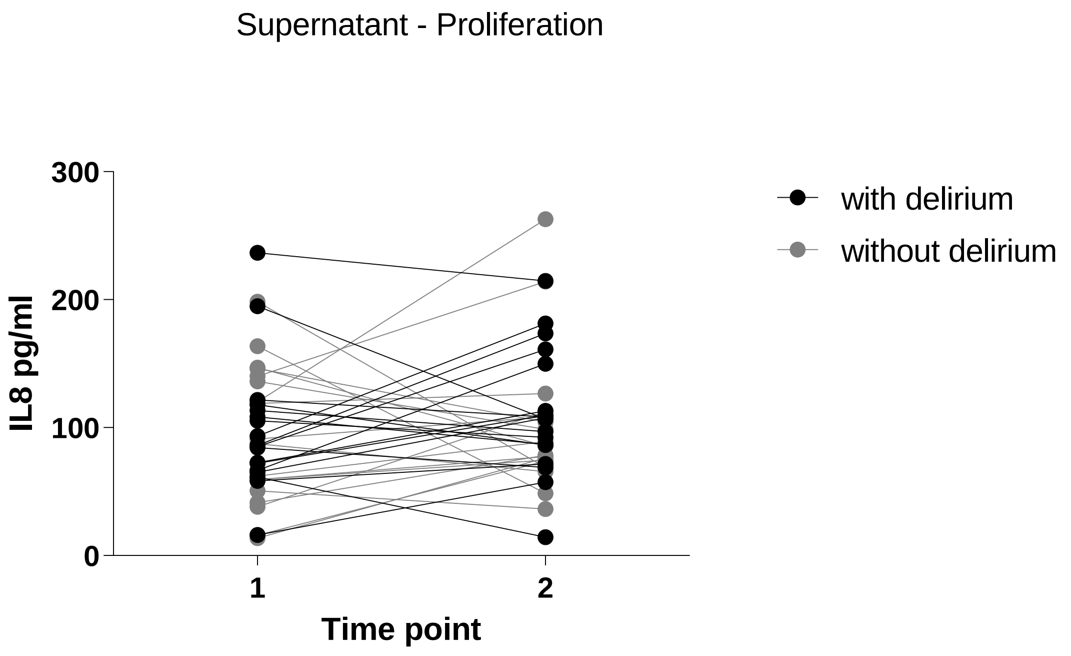
Supernatant - Proliferation
IL8 pg/ml
300
200
100
0
1
2
Time point
with delirium
without delirium

o)

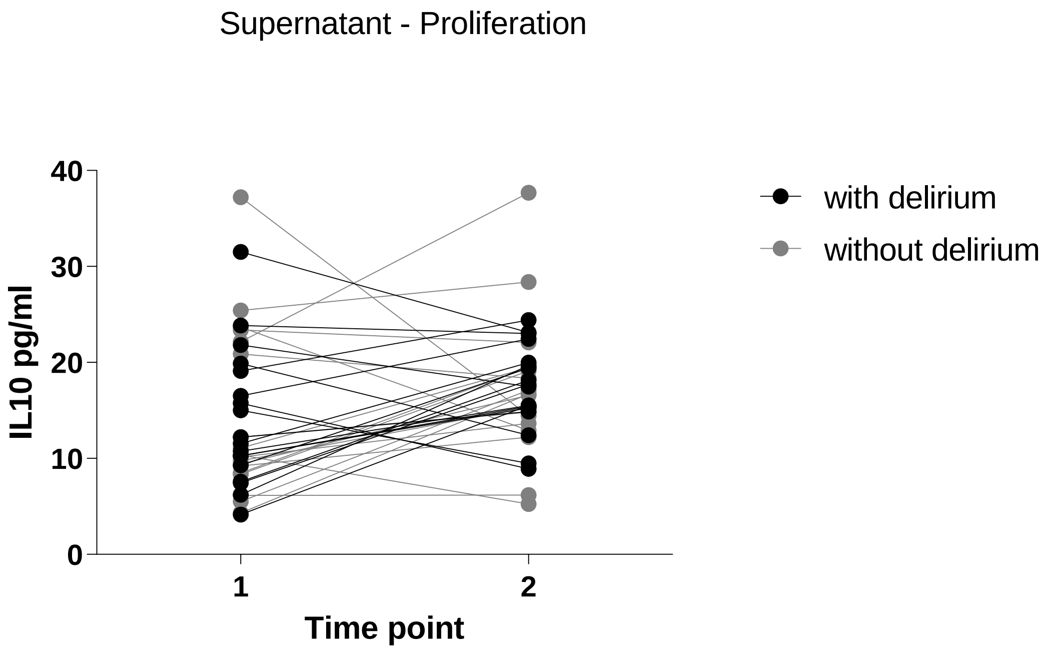
Supernatant - Proliferation
40
30
20
10
0
IL10 pg/ml
1
2
Time point
with delirium
without delirium

p)

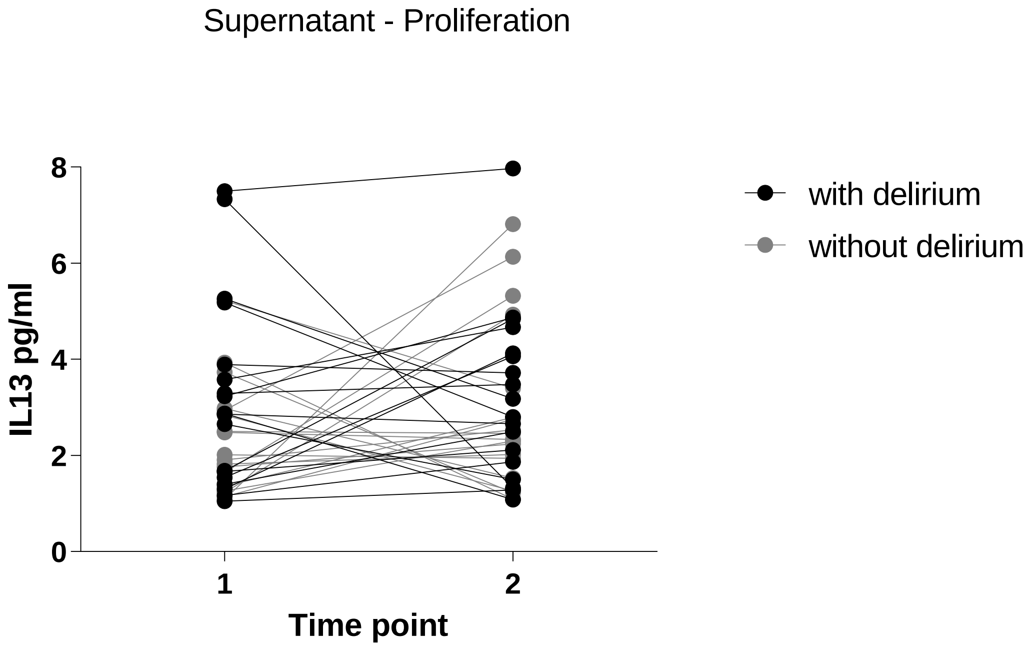
Supernatant - Proliferation
8
6
4
2
0
IL13 pg/ml
1
2
Time point
with delirium
without delirium

q)

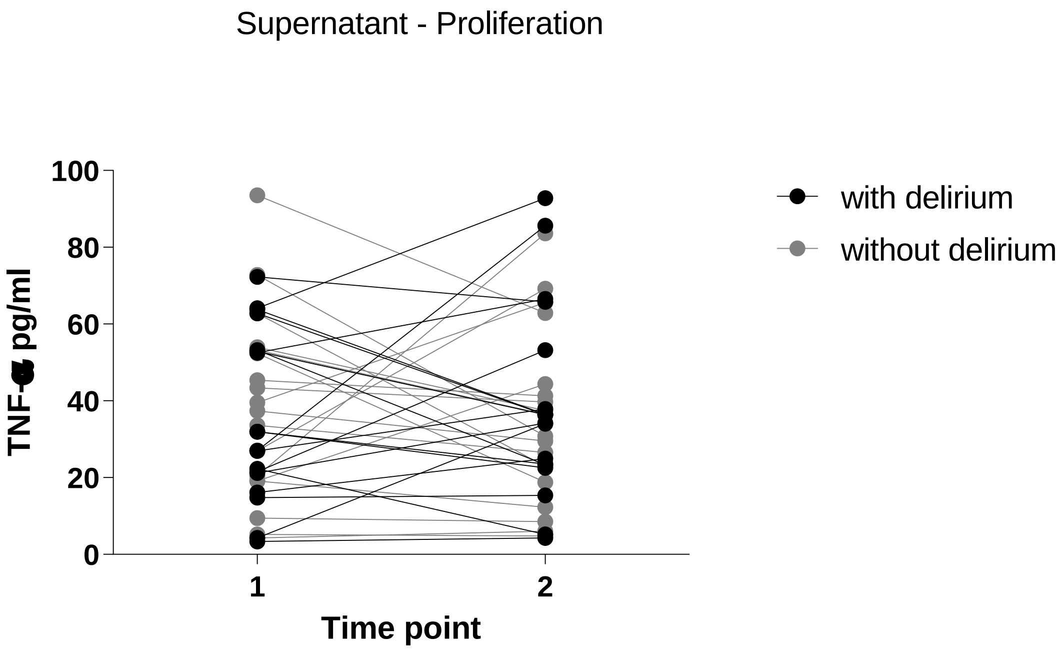
Supernatant - Proliferation
TNF-α pg/ml
100
80
60
40
20
0
1
2
Time point
with delirium
without delirium

r)

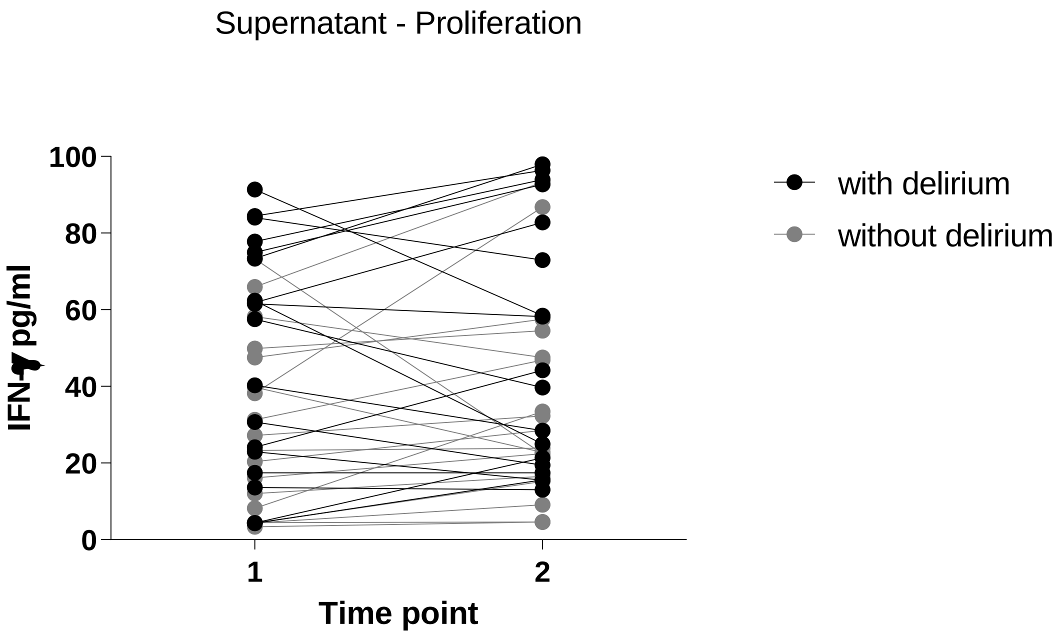
Supernatant - Proliferation
IFN-γ pg/ml
100
80
60
40
20
0
1
2
Time point
with delirium
without delirium

s)

Supernatant - Differentiation

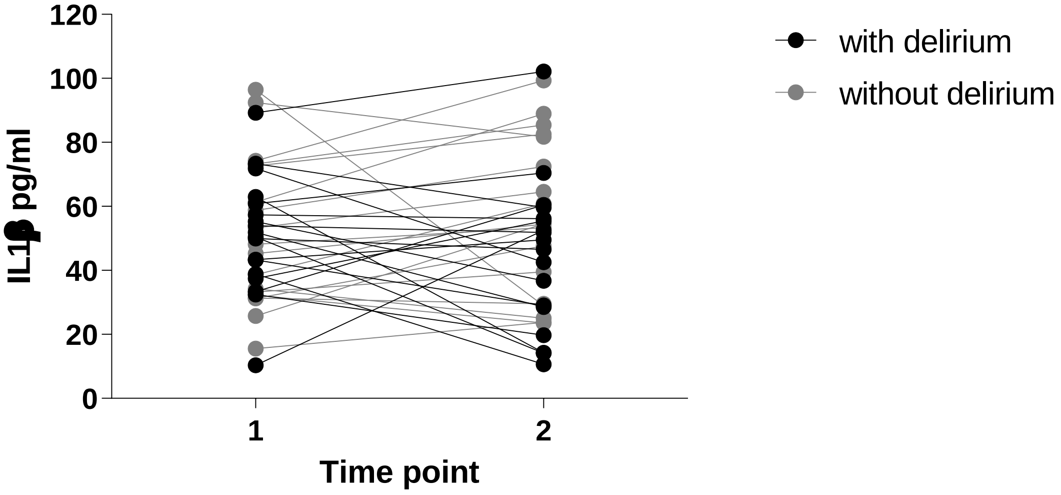

t)

Supernatant - Differentiation

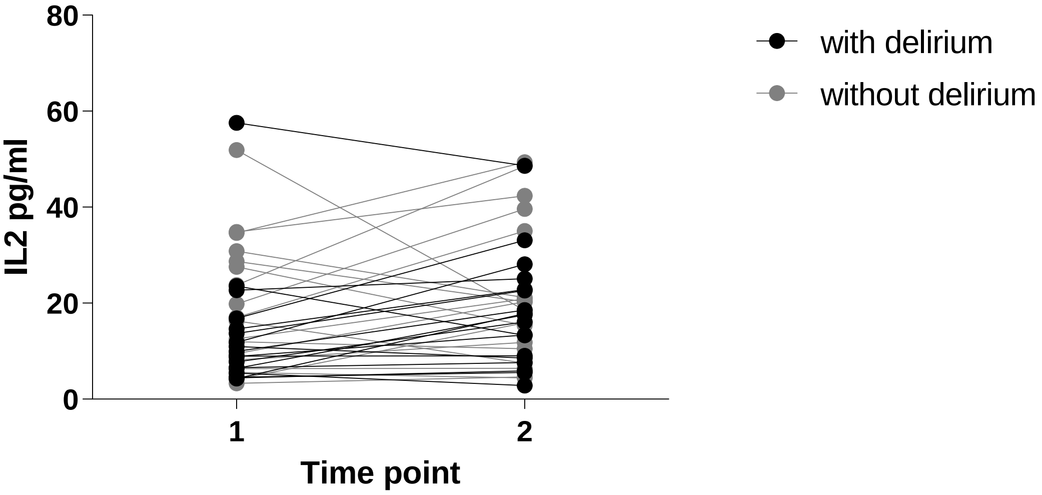

u)

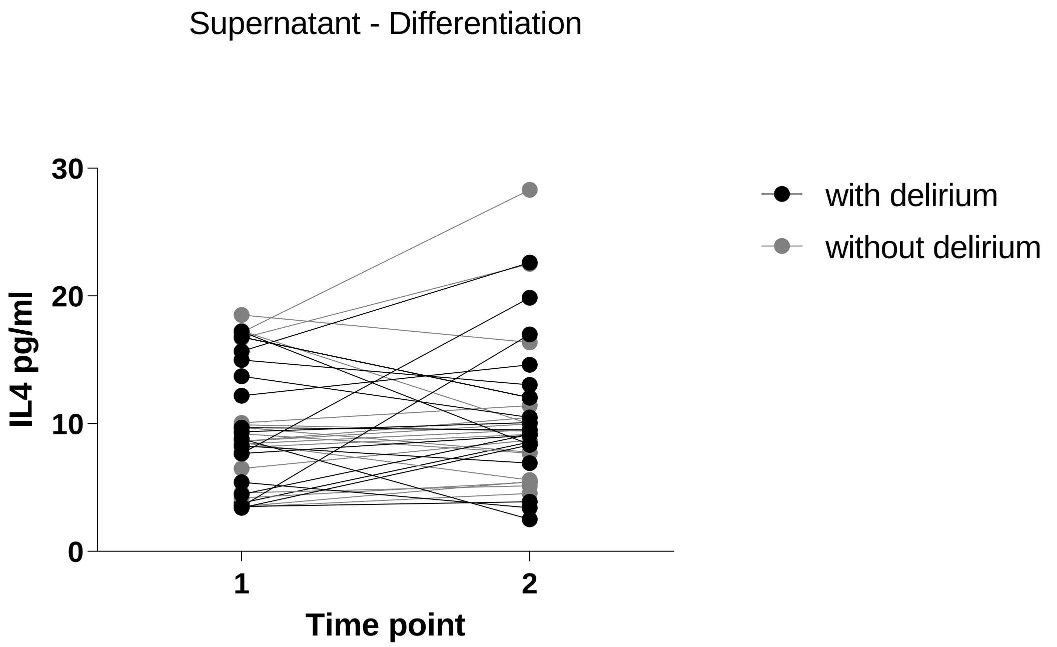
Supernatant - Differentiation
IL4 pg/ml
30
20
10
0
1
2
Time point
with delirium
without delirium

v)

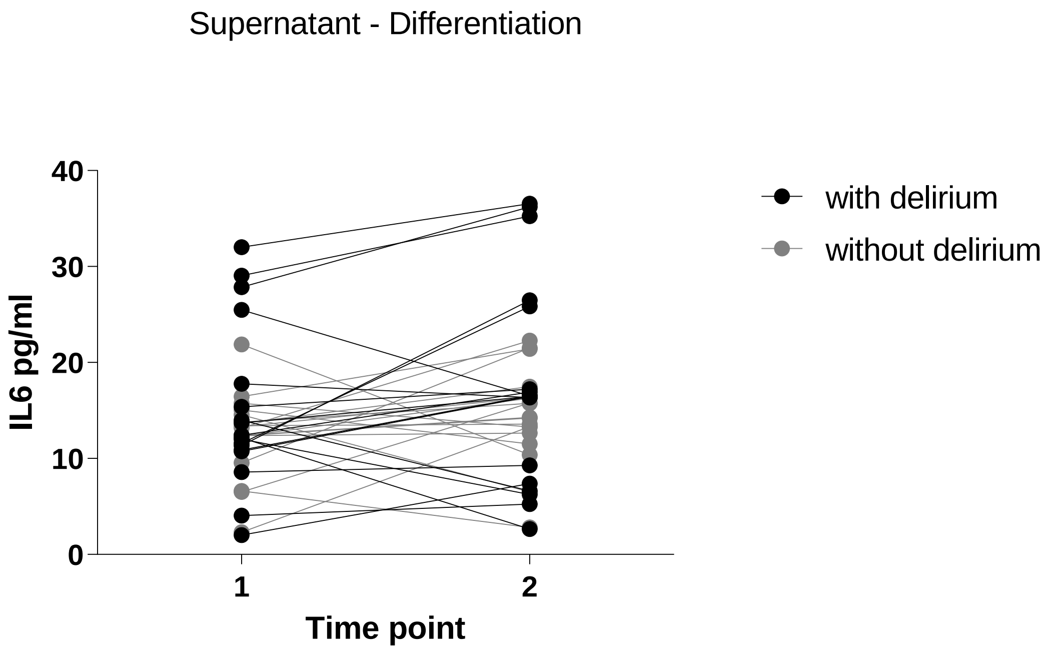
Supernatant - Differentiation
IL6 pg/ml
40
30
20
10
0
1
2
Time point
with delirium
without delirium

w)

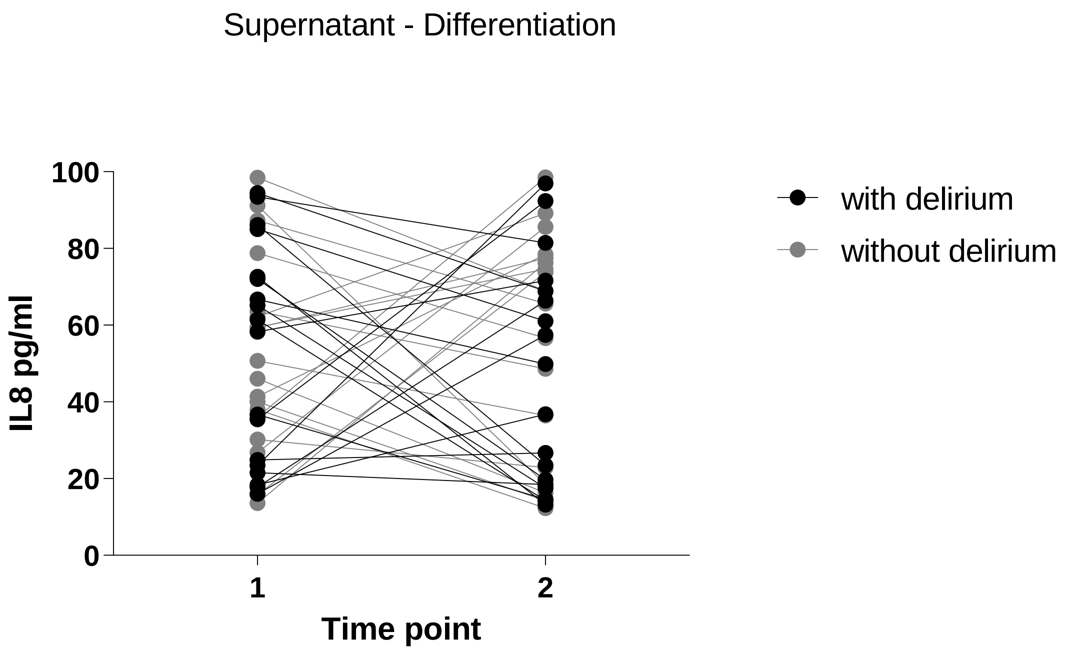
Supernatant - Differentiation
IL8 pg/ml
100
80
60
40
20
0
1
2
Time point
with delirium
without delirium

x)

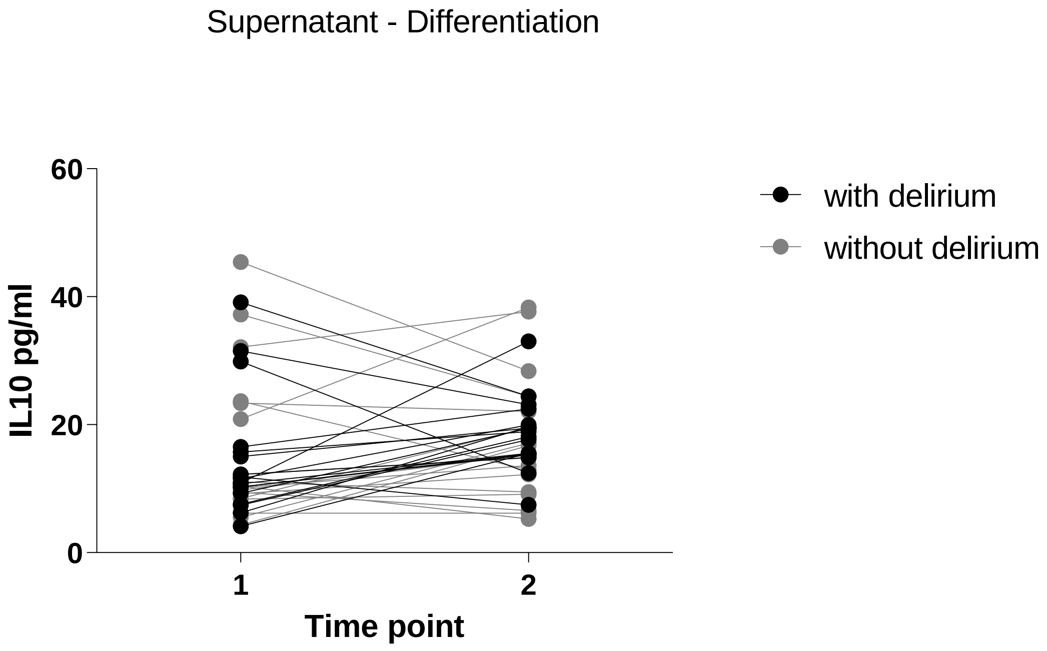
Supernatant - Differentiation
IL10 pg/ml
60
40
20
0
1
2
Time point
with delirium
without delirium

y)

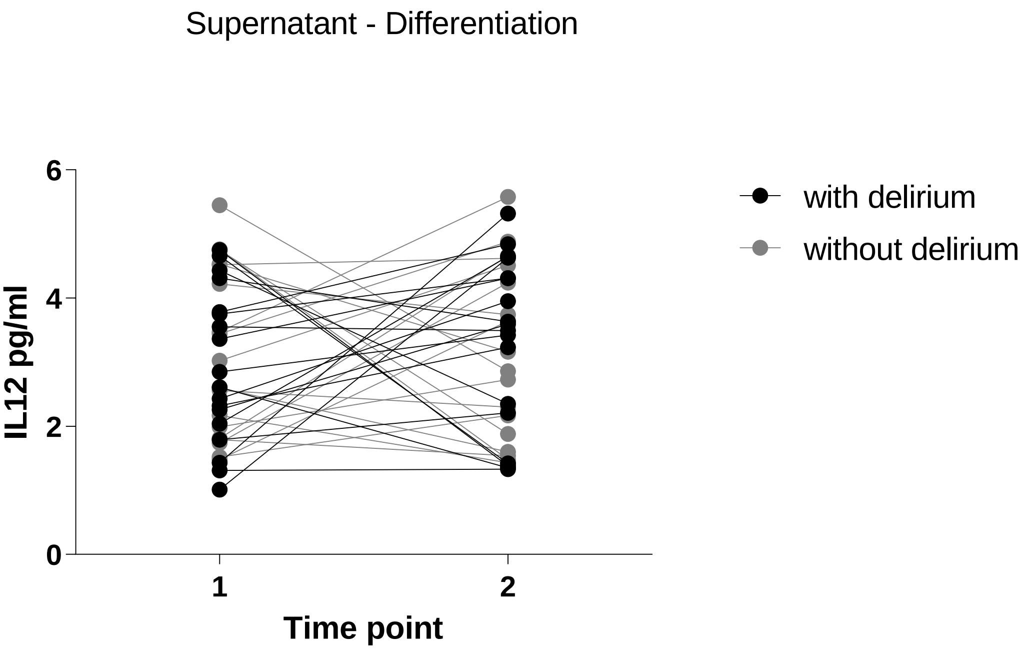
Supernatant - Differentiation
IL12 pg/ml
6
4
2
0
1
2
Time point
with delirium
without delirium

z)

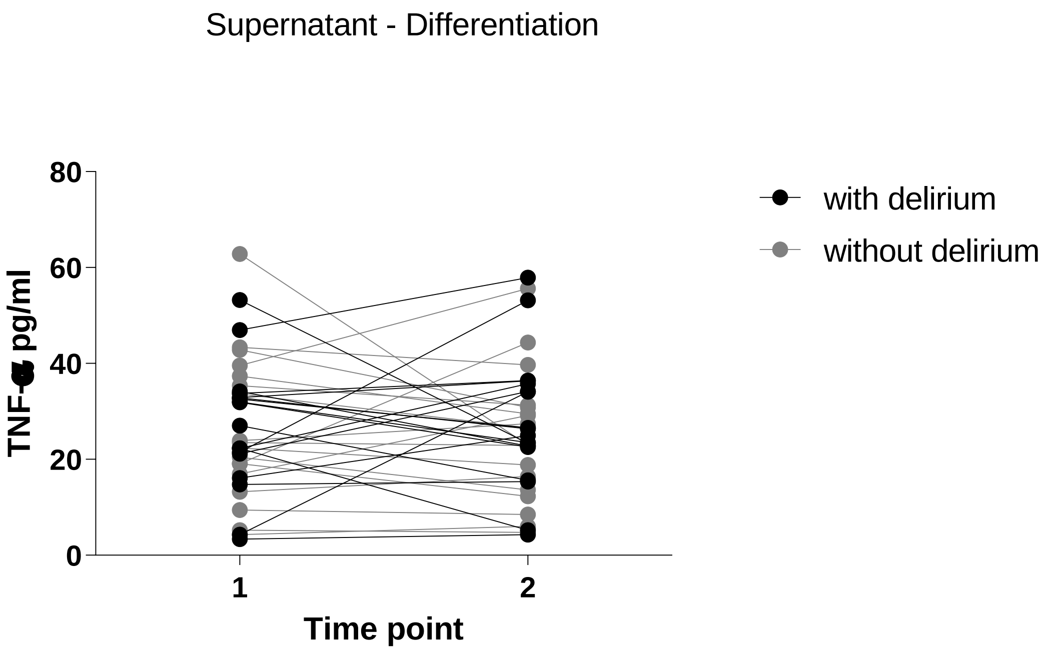
Supernatant - Differentiation
TNF-α pg/ml
80
60
40
20
0
1
2
Time point
with delirium
without delirium

zz)

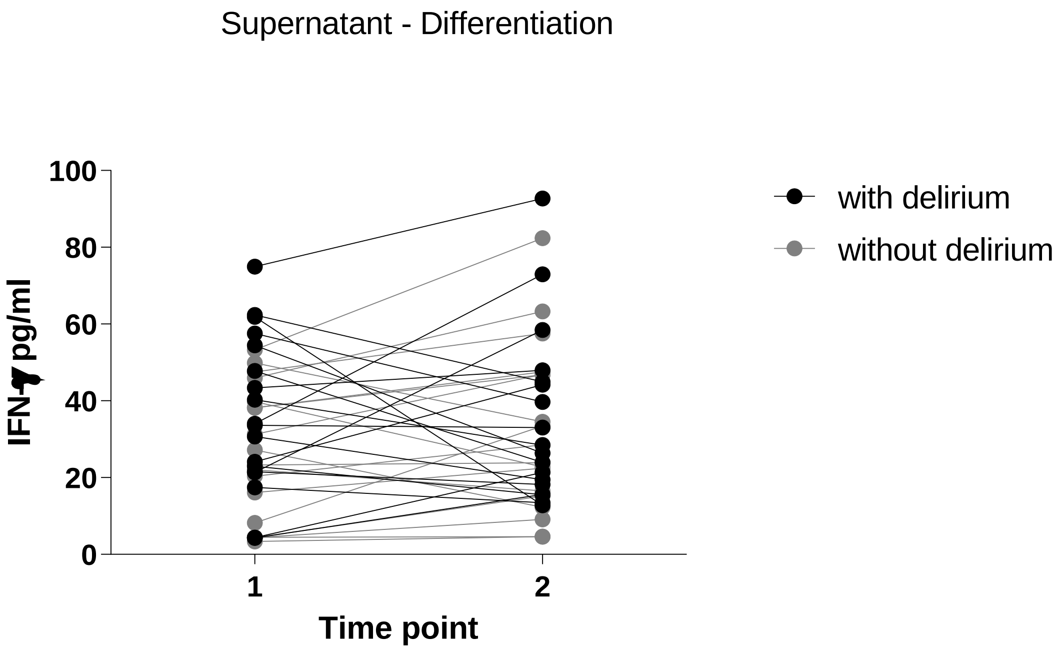
Supernatant - Differentiation
IFN-γ pg/ml
100
80
60
40
20
0
1
2
Time point
with delirium
without delirium
