# Supplementary material for: Neurogenesis is disrupted in human hippocampal progenitor cells upon exposure to serum samples from hospitalized COVID-19 patients with neurological symptoms
Source: Mol Psychiatry. 2022 Oct 5;27(12):5049–61. doi: 10.1038/s41380-022-01741-1 (PMC9763123; doi:10.1038/s41380-022-01741-1)
Supplement: Supplementary file 6 — Supplementary Legends [file 41380_2022_1741_MOESM6_ESM.pdf]

Supplementary Figure 1. Immunostaining images of proliferating, neurogenic and apoptotic markers in vehicle conditions and in treated conditions. **(a-h)** Cells were exposed to media (without serum) for 2 days during proliferation, and markers of stemness, Sox2 (green) and Nestin (red), as well as markers of proliferation KI67 (green) and apoptosis CC3 (red) were detected over the total number of cells DAPI (blue). After 2 days of proliferation, cells were left to differentiate (again without serum) for additional 4 days and neuroblasts and mature neurons, DCX (red) and Map2 (green), astrocytes, S100β (green), and again apoptotic cells, CC3 (red) were detected over the total number of cells DAPI (blue). **(i-m)** Cells were exposed to media (with serum collected at Time point 1) for 2 days during proliferation, and markers of proliferation KI67 (green) and apoptosis CC3 (red) were detected over the total number of cells DAPI (blue). After 2 days of proliferation, cells were left to differentiate (again with serum collected at Time point 1) for additional 4 days and neuroblasts and mature neurons, DCX (red) and Map2 (green), and apoptotic cells, CC3 (red) were detected over the total number of cells DAPI (blue). 10x magnification.

Supplementary Figure 2. Cell treatment with serum samples from COVID-19 patients with delirium did not change the expression of markers of stemness (during proliferation) or marker of astrocytes (during differentiation), when comparing the two groups (delirium vs non-delirium) and the two time points. (a-b) Cell treatment with serum samples from patients with delirium did not change the expression of markers of stemness (Sox2+, Nestin+ cells), when comparing the two groups (delirium vs non-delirium) and the two time points. **(c)** Cell treatment with serum samples from patients with delirium did not change the expression of markers of astrocytes (S100β), when comparing the two groups (delirium vs non-delirium) and the two time points. Two-way mixed ANOVA was performed.

Supplementary Figure 3. Levels of cytokines detected in serum samples and cell supernatant, both at proliferation and differentiation stage, which did not differ between COVID-19 patients with and without delirium. (a-i) Concentrations of candidate cytokines in serum samples of patients with and without delirium across Time point 1 and 2. **(j-r)** Concentrations of candidate cytokines, during proliferation, in cell supernatant of cells exposed to treatment with serum samples form patients with and without delirium across Time point 1 and 2. **(s-zz)** Concentrations of candidate cytokines, during differentiation, in cell supernatant of cells exposed to treatment with serum samples form patients with and without delirium across Time point 1 and 2. Two-way mixed ANOVA was performed.

Supplementary Figure 4. Treatment with an antibody against IL6, during proliferation, did not prevent the detrimental effect of serum from COVID-19 patients with delirium on both cell neurogenesis and apoptosis. (a-f) Cell treatment with IL6A (0.1ug/ml), during proliferation, did not prevent the decrease in neurogenesis (DCX+ and Map2+ cells) and increase in apoptosis (CC3+cells) previously observed upon treatment with serum samples from patients with delirium. This is confirmed across treatment with serum samples collected at Time point 1 and 2. (**g-l**) Cell treatment with IL6A, during both proliferation and differentiation, did not prevent the decrease in neurogenesis (DCX+ and Map2+ cells) and increase in apoptosis (CC3+cells) previously observed upon treatment with serum samples from patients with delirium. Again, this is confirmed across treatment with serum samples collected at Time point 1 and 2. Two-way mixed ANOVA with Bonferroni's post hoc test was performed. Data are shown as mean; ****$p<0.0001$ comparisons as indicated.

Supplementary Figure 5. Treatment with recombinant IL12 or IL13, at concentrations previously found in supernatant of cells exposed to serum from patients with delirium, decrease cell proliferation, neurogenesis and increase apoptosis, whereas co-treatment with the JAKi baricitinib, ruxolitinib or tofacitinib prevent these detrimental effects. (a-b) Cell treatment with IL12 with delirium (20pg/ml) was able to cause a decrease in cell proliferation (Ki67) and increase in apoptosis (CC3), when compared with IL12 without delirium (3pg/ml). Interestingly, co-treatment with baricitinib, ruxolitinib or tofacitinib (all 1nM) were able to prevent these detrimental effects. (**c-e**) Cell treatment with IL13 with delirium (25pg/ml) was able to cause a decrease in neurogenesis (DCX and Map2) and increase in apoptosis (CC3), when compared with IL13 without delirium (4pg/ml). Interestingly, co-treatment with baricitinib, ruxolitinib or tofacitinib (all 1nM) were able to prevent

these detrimental effects. Two-way mixed ANOVA with Bonferroni's post hoc test was performed. Data are shown as mean; ***$p<0.001$, ****$p<0.0001$ comparisons versus vehicle or as indicated.
